# DELirium treatment with Transcranial Electrical Stimulation (DELTES): study protocol for a multicentre, randomised, double-blind, sham-controlled trial

**DOI:** 10.1136/bmjopen-2024-092165

**Published:** 2024-11-02

**Authors:** Julia van der A, Yorben Lodema, Thomas H Ottens, Dennis J L G Schutter, Marielle H Emmelot-Vonk, Willem de Haan, Edwin van Dellen, Indira Tendolkar, Arjen J C Slooter

**Affiliations:** 1 Department of Intensive Care Medicine and University Medical Center Utrecht Brain Center, University Medical Centre Utrecht, Utrecht, The Netherlands; 2 Department of Psychiatry and University Medical Center Utrecht Brain Center, University Medical Centre Utrecht, Utrecht, The Netherlands; 3 Intensive Care Unit, HagaZiekenhuis, Den Haag, The Netherlands; 4 Universiteit Utrecht, Utrecht, The Netherlands; 5 Department of Geriatrics, University Medical Centre Utrecht, Utrecht, The Netherlands; 6 Alzheimer Center and Department of Neurology, Amsterdam Neuroscience, VU Medisch Centrum, Amsterdam, The Netherlands; 7 Department of Neurology and Vrije Universiteit Brussel, UZ Brussel, Brussel, Belgium; 8 Donders Institute for Brain, Cognition and Behavior, Department of Psychiatry, Radboud Universiteit, Nijmegen, The Netherlands

**Keywords:** Delirium, Electroencephalography, Randomized Controlled Trial

## Abstract

**Introduction:**

Delirium, a clinical manifestation of acute encephalopathy, is associated with extended hospitalisation, long-term cognitive dysfunction, increased mortality and high healthcare costs. Despite intensive research, there is still no targeted treatment. Delirium is characterised by electroencephalography (EEG) slowing, increased relative delta power and decreased functional connectivity. Recent studies suggest that transcranial alternating current stimulation (tACS) can entrain EEG activity, strengthen connectivity and improve cognitive functioning. Hence, tACS offers a potential treatment for augmenting EEG activity and reducing the duration of delirium. This study aims to evaluate the feasibility and assess the efficacy of tACS in reducing relative delta power.

**Methods and analysis:**

A randomised, double-blind, sham-controlled trial will be conducted across three medical centres in the Netherlands. The study comprises two phases: a pilot phase (n=30) and a main study phase (n=129). Participants are patients aged 50 years and older who are diagnosed with delirium using the Diagnostic and Statistical Manual of Mental Disorders, Fifth Edition, Text Revision criteria (DSM-5-TR), that persists despite treatment of underlying causes. During the pilot phase, participants will be randomised (1:1) to receive either standardised (10 Hz) tACS or sham tACS. In the main study phase, participants will be randomised to standardised tACS, sham tACS or personalised tACS, in which tACS settings are tailored to the participant. All participants will undergo daily 30 min of (sham) stimulation for up to 14 days or until delirium resolution or hospital discharge. Sixty-four-channel resting-state EEG will be recorded pre- and post the first tACS session, and following the final tACS session. Daily delirium assessments will be acquired using the Intensive Care Delirium Screening Checklist and Delirium Observation Screening Scale. The pilot phase will assess the percentage of completed tACS sessions and increased care requirements post-tACS. The primary outcome variable is change in relative delta EEG power. Secondary outcomes include (1) delirium duration and severity, (2) quantitative EEG measurements, (3) length of hospital stay, (4) cognitive functioning at 3 months post-tACS and (5) tACS treatment burden. Study recruitment started in April 2024 and is ongoing.

**Ethics and dissemination:**

The study has been approved by the Medical Ethics Committee of the Utrecht University Medical Center and the Institutional Review Boards of all participating centres. Trial results will be disseminated via peer-reviewed publications and conference presentations.

**Trial registration number:**

NCT06285721.

**STRENGTHS AND LIMITATIONS OF THIS STUDY**

- This is a randomised, double-blind, sham-controlled trial to evaluate transcranial alternating current stimulation (tACS) as treatment for delirium.
- The analysis of electroencephalography (EEG) before and after tACS will provide insights into the neurophysiological effects of tACS in delirium.
- An inital pilot phase will assess the feasibility of tACS in a delirium population.
- This study incorporates a personalised treatment arm that tailors tACS settings to an individual participant.
- Applicability to hyperactive delirium may be limited due to the requirement for patients to complete EEG assessments.

## Introduction

 Delirium, a neuropsychiatric syndrome characterised by an acute disturbance in consciousness and cognition precipitated by a medical condition such as infection or surgery, affects approximately 23% of medical inpatients.^1 2^ It is associated with extended hospitalisation, long-term cognitive dysfunction, increased mortality and increased healthcare costs.^3^,^8^ There is no specific treatment for delirium itself. Current management strategies primarily target precipitating factors and employ (non-)pharmacological interventions to alleviate symptoms.^1 9^ As duration of delirium is independently associated with worsened long-term cognitive outcomes and dementia, interventions to treat delirium itself are needed.^4 10 11^

Delirium is one of the clinical manifestations of acute encephalopathy, a rapidly developing pathobiological process in the brain,^12^ measurable by electroencephalography (EEG). EEG power spectral analysis in patients with acute encephalopathy presenting as delirium consistently shows increased power in delta and theta bands, primarily in frontal regions, and reduced power in the alpha band, predominantly in occipital and parietal regions.^13^,^19^ Of these changes, reduced relative delta power (0.5–4 Hz) is the most robust feature and can be used to classify the presence of delirium based on EEG compared with non-delirious control patients.^20 21^ This shift to slow wave activity correlates with delirium severity, strengthening the evidence for a relation between these phenomena.^19^ Furthermore, delirium is associated with decreased functional brain connectivity and reduced network efficiency in the alpha frequency band.^16 17^ Studies using functional MRI have demonstrated decreased integration and efficiency of the default mode network (DMN) in patients with postoperative delirium.^22 23^ Another study showed that network alterations persist after 3 months and correlate with cognitive impairment, indicating an association between connectivity changes and cognitive outcomes.^24^

Recent studies in healthy individuals have demonstrated the potential of transcranial alternating current stimulation (tACS) in modulating brain activity by entrainment of specific cortical rhythms based on the applied stimulation frequency.^25^,^27^ The administration of tACS is suggested to phase-lock large populations of neurons in the superficial layers of the cerebral cortex, inducing neural synchronisation in the corresponding frequency, and changing brain connectivity.^28 29^ Studies on healthy individuals have revealed that tACS applied in the alpha frequency range can augment alpha activity and functional brain connectivity,^2530^,^33^ both affected during delirium.^34^ Furthermore, a meta-analysis has indicated a clear beneficial effect of tACS on cognition in other populations, including improvements in attention and working memory,^35^ which are cognitive domains also affected during delirium.^1^ Interestingly, a recent study with healthy volunteers showed that alpha-tACS not only augments alpha activity but also strengthens connectivity within the DMN,^25^ the primary network disturbed during delirium.^22 23^ Additionally, oscillatory entrainment can have cross-frequency effects,^30 36^ meaning that tACS applied within the alpha frequency range can lead to a decrease in relative delta power. Taken together, tACS might be able to reduce delta activity, reinforce alpha activity and connectivity in brain regions that show altered connectivity during delirium,^22 23^ potentially offering therapeutic benefits.

When applying tACS as a potential treatment for delirium, the most straightforward approach is to apply tACS in the alpha frequency range, targeting both reduced alpha power and functional connectivity seen in delirium.^15^,^18^ However, numerous approaches in terms of stimulation location and frequency are possible, which might be equally or more effective in treating delirium than alpha-tACS. Incorporating functional brain connectivity changes of individual patients into personalised treatment could improve treatment effectiveness, reduce adverse effects, decrease the need for trial and error in clinical trials and enhance our understanding of the mechanisms underlying treatment effects.^37^ The use of computational models may allow one to infer how modifications of neuronal properties might influence emergent neuronal activity and treatment response.^38^ A promising type of computational model is the neural mass model, which models brain activity of large populations of neurons.^39^ Using a network of coupled neural masses, neuronal activity similar to an encephalopathic EEG has been simulated.^40^ Building on this, this study will apply neural mass modelling of individual functional connectivity changes in a virtual trial to optimise treatment settings.

In the current trial, we will evaluate whether tACS normalises brain activity, specifically relative delta power, in delirium. To date, no randomised controlled trials (RCTs) investigated tACS as treatment for delirium, highlighting significant gaps in our understanding of the feasibility, effectiveness and the most effective application strategies. Therefore, the trial will begin with a pilot phase aimed to assess feasibility. On successful completion of the pilot phase, the main study phase will commence with three study arms to assess the efficacy of tACS in reducing relative delta power: a standardised treatment arm, a sham control arm and a personalised treatment arm based on a computational model and virtual trial. We hypothesise that both standardised and personalised tACS will decrease relative delta power compared with sham tACS in delirium patients. By adopting this two-step approach, this study aims to evaluate the feasibility as well as the effectiveness of tACS in patients with delirium.

## Methods and analysis

### Study objectives

For the pilot phase, the primary objective is to evaluate the safety and tolerability of tACS in patients with delirium. The main study phase aims to determine the efficacy of a single session of standardised or personalised tACS in reducing EEG relative delta power in patients with delirium. Secondary objectives include assessment of the impact of daily standardised or personalised tACS compared with sham on the duration and/or severity of delirium, the length of hospital stay and cognitive functioning 3 months after the initial tACS session.

### Study design and setting

This study is a double-blind, RCT conducted across three medical centres in the Netherlands: the University Medical Center (UMC) Utrecht, Radboud UMC and HagaZiekenhuis. To assess safety and feasibility of tACS in delirious patients, the study will start with a pilot phase in which 30 patients will be randomised in a 1:1 ratio to receive daily either standardised active tACS or sham treatment for a maximum of 14 days, or until resolution of delirium or hospital discharge.

**Figure 1:**
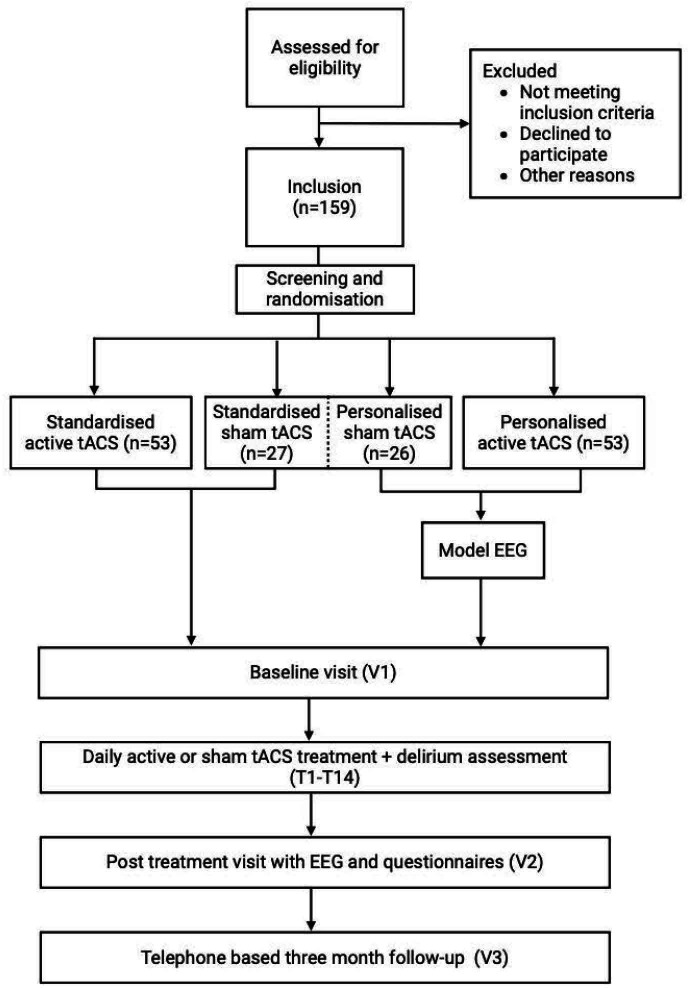
Study flow chart. EEG, electroencephalography; tACS, transcranial alternating current stimulation; T, treatment; V, visit.

On completion of the pilot phase, the main study phase will begin, introducing the personalised treatment arm. Criteria for continuing to the main study phase are defined under outcomes. All patients from the pilot phase will be included in the main study analyses. Randomisation weights will be recalculated, and participants will be allocated in an overall 1:1:1 ratio to receive either standardised tACS, personalised tACS, or sham treatment (ie, combining personalised sham and standardised sham tACS into one arm). The baseline visit will include delirium assessment using the Delirium Interview,^41^ administered by a trained researcher, and reviewed by an expert delirium panel. Furthermore, information will be collected from the electronic patient record and the Clinical Frailty Scale^42^ will be evaluated. After these assessments, the first treatment session starts which includes a 64-channel EEG measurement before and after the first tACS or sham treatment. Also, a questionnaire on sensation to assess possible adverse events (AEs) of tACS, a questionnaire on feasibility and questionnaire on blinding and subjective treatment experiences (online supplemental appendix 1) will be administered. Following this, daily tACS or sham treatment visits and delirium assessments will take place for a maximum of 14 days, or until resolution of delirium or hospital discharge, whichever comes first. To account for fluctuations in delirium symptoms, resolution of delirium is defined as two consecutive negative delirium assessments. The treatment phase will end with a close-out visit including a follow-up 64-channel EEG and administration of the questionnaires on sensation, blinding and subjective treatment experiences. A brief cognitive assessment using the Telephone Interview for Cognitive status, modified version (TICS-M)^43 44^ is planned at 3 months after the first tACS session. The study design is illustrated in figure 1, and the study schedule is presented in table 1.

**Table 1:** Overview study procedures

Procedures (time needed)	Baseline visit (V1)	First treatment visit (T1)	Additional treatment visits (T2 up to T14)	Post-treatment visit (V2)	Follow-up visit (V3)
Medical history*	X				
Physical health*	X				X
Current medication use*	X	X	X	X	X
Clinical Frailty Scale*	X				
Sensation questionnaire (5 min)		X		X	
Blinding and subjective treatment experience (1 min)		X		X	
Feasibility questionnaire (5 min)		X			
Delirium interview (10 min)	X				
ICDSC (10 min)	X	X	X	X	
DOSS (5 min)†	X	X	X	X	
TICS-M (10 min)					X
EEG (40 min)	X‡	X		X	
tACS (30 min)		X	X		
Estimated total duration	25 or 60 min‡	95 min	45 min	65 min	15 min

* This information will be recorded as part of standard clinical care, and missing information will be requested via family and will therefore not require additional time.

† Only non-intensive care unit () patients will be assessed using the DOSS.

‡ Only the group randomised to personalised tACS will receive an EEG during the baseline visit.

DOSS, Delirium Observation Screening Scale; EEG, ElectroencephalogramICDSC, Intensive Care Delirium Screening Checklist; tACStranscranial altering current stimulationTICS-M, Telephone Interview for Cognitive status, modified version

### Sample size and statistical power

The sample size calculation is based on data obtained from a previous study that examined EEG findings in both delirious and non-delirious patients.^15^ In this study, patients with delirium showed a median relative delta power of 0.59 (IQR 0.47–0.71), while those without delirium had a median of 0.20 (IQR 0.17–0.26), resulting in an effect size of 0.39 (0.20–0.59). This study excluded patients in whom the diagnosis delirium was not certain, which may have inflated the effect size. It is therefore anticipated that both standardised and personalised tACS will lead to a more modest decrease of 0.15 in relative delta EEG power poststimulation compared with prestimulation measurements. We hypothesise that personalised tACS may be superior to standardised tACS in reducing relative delta power. However, the lack of data to support this claim necessitates assuming equal effectiveness for both arms in the sample size calculation. Based on these assumptions, a sample size of 159 participants (ie, 53 per group) was estimated using G*Power 3.1. This estimation considered an effect size of 0.15 with a SD of 0.3, an alpha of 0.05 and 80% statistical power. Patients who do not complete the initial tACS session with EEG recordings will be replaced, as well as patients who withdraw consent.

### Study population

In total, 159 patients aged 50 years or older with a diagnosis of delirium will be included in the study.

#### Inclusion criteria for eligibility

In order to be eligible to participate in this study, a participant must meet all of the following inclusion criteria:

- Age over 50 years.
- Diagnosis of delirium.
- Richmond Agitation and Sedation Scale (RASS)^45^ score of −2 to +2.
- Delirium duration of at least 2 days prior to study inclusion, based on delirium assessments and/or descriptions in the medical and/or nursing files.
- Causes underlying delirium are being treated adequately, as assessed by the treating physician and a panel of delirium experts (ie, psychiatrist, geriatrician and intensivist).

#### Exclusion criteria for eligibility

A potential participant who meets one or more of the following criteria will be excluded from participation in this study:

- Inability to conduct valid delirium screening assessment (eg, deaf, blind) or inability to speak Dutch or English.
- A moribund state.
- Alcohol/substance abuse withdrawal or stroke as the presumed cause of delirium.
- Diagnosis of dementia, based on medical record review and/or a score of ≥4.5 on the short form of the Informant Questionnaire on Cognitive Decline in the Elderly.^46^
- Brain injury of any type (eg, traumatic, vascular, post anoxic) in the previous 6 weeks.
- One or more contraindications for tACS:
  - Large or ferromagnetic metal parts in the head (except for a dental wire).
  - Implanted cardiac pacemaker or neurostimulator.
  - Skin disease or inflammation at the stimulation sites.
  - History of epilepsy.

#### Inclusion criteria for randomisation

- All inclusion criteria are met.
- Diagnosis of delirium is confirmed using the Delirium Interview^41^ and consultation with a delirium expert who is part of the research team (psychiatrist, geriatrician and/or intensivist).
- Written informed consent obtained from legal representative.

#### Patient withdrawal

If a patient and/or legal representative wants to withdraw from the study, they can do so without any consequences. We will adhere to the definitions and guidelines stipulated in the code of conduct relating to the expression of objection by incapacitated (psycho)geriatric patients in the context of the WMO (2002). The clinician or investigator can decide to withdraw a subject from the study for urgent medical reasons. There are no expected negative effects of prematurely ending the stimulation sequence.

### Informed consent, randomisation and blinding

For surgical patients, a flyer is provided during the preoperative screening to inform patients and their legal representatives about the study, enabling them to familiarise themselves with this study in advance and consider participation in the event of delirium occurrence. In non-surgical patients, this flyer is provided to the wards with the request to hand this to newly admitted patients. Consultants, including psychiatrists, geriatricians and neurologists, and ward physicians are asked to screen for potential participants. On identification of patients eligible to participate in the trial, consultants and ward physicians inform the research team. The research team will inform the patient and their legal representatives about the study. If the patient and legal representative are possibly willing to participate, the investigator provides the information letter and provides them at least 1 day to consider study participation. If a patient is eligible for study participation, initial informed consent will be obtained from a legal representative, as the patient may be unable to provide consent when delirious (see online supplemental appendix 2 for an example of the consent form). Legal representation is identified using a hierarchical model consistent with local and national laws and regulations. Once patients regain capacity to provide informed consent, they will be asked to provide written informed consent themselves. At any time, the patient or their legal representatives can refuse or withdraw consent for the study without providing a reason and without impacting the treatment provided.

Delirious patients who meet all inclusion criteria but none of the exclusion criteria for eligibility and randomisation will be randomised to one of the study arms. Randomisation will be conducted electronically via the Castor Electronic Data Capture (EDC) study management system (Castor, Ciwit B.V., Amsterdam, the Netherlands), using a validated block randomisation model, stratified by study centre. In the pilot phase, standardised active tACS and sham carry equal weight (1:1). Patients will be randomised with block sizes of 2 and 4. In the main study phase, four groups will be created in Castor EDC (standardised active, personalised active, standardised sham, personalised sham) with different weights, depending on the number of participants who have been randomised to the active and standardised sham groups during the pilot phase. As the first 30 patients are included during the pilot phase, randomisation weights will be 3, 4, 1 and 2, respectively. These numbers are chosen to closely match the overall 1:1:1 allocation. Randomisation will be performed with block sizes of 10 and 20, which are randomly selected.

Following randomisation, a designated study team member not involved in any other study procedures or data analysis will be aware of the randomisation outcome. This person will have access to a list of codes that permits the tACS device to deliver active or sham stimulation. Participants and all other study staff will be blinded to whether active or sham stimulation is applied. To ensure blinding during the intervention, the monitor displaying the raw ECG traces will be covered with cardboard paper before the start of the procedure for patients on continuous ECG monitoring. Due to the additional EEG required in the personalised treatment arm, blinding with regard to receiving standardised or personalised tACS will not be possible. However, EEG preprocessing and data analysis will be performed blinded for treatment allocation.

### Intervention

tACS will be administered to participants who are randomised using the Nurostym tES device (Brainbox, UK) by a trained member of the study team. The same tACS device and settings will be used across all three participating centres to ensure consistency of results. For all study arms, tACS will be administered at an intensity of 2.0 mA (peak to peak) for 30 min using two 5×5 cm saline-soaked electrodes while the impedance is kept below 10 kΩ. Electrode placement (described below) follows the 10–10 EEG system, ensuring consistent positioning of tACS electrodes across different stimulation days, patients and centres. The electrodes will be positioned beneath a 64-channel EEG cap. During the first treatment session, this cap will also be used for repeated EEG measurements, whereas on subsequent treatment days, it will serve solely as a reference for tACS electrode positioning. Treatment with psychoactive medication(s) that is deemed necessary for the participant will be continued as prescribed by the treating physician.

#### Standardised tACS

**Figure 2:**
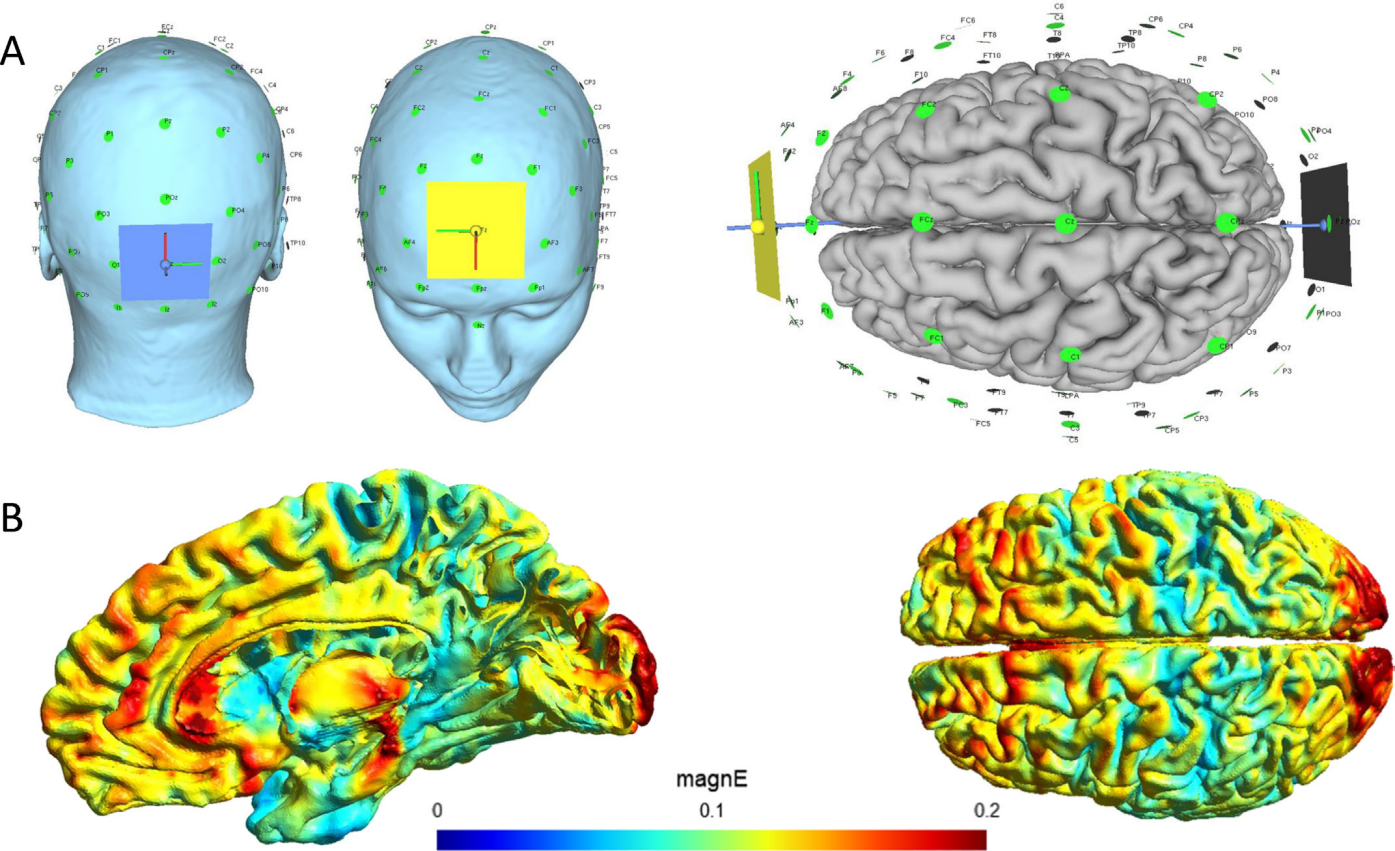
Standardised approach for applying transcranial alternating current stimulation (tACS). (A) Representation of the electrode placement. Two 5×5 cm electrodes will be positioned over AFz (anterior) and Oz (posterior) locations, indicated by coloured squares (blue for posterior, yellow for anterior). (B) Visualisation of the electric field distribution in the brain during tACS with an intensity of 2 mA (peak to peak). The colour map represents the magnitude of the electric field (magnE), measured in volts per metre (V/m). SimNIBS software (version 4) was used for simulation.^56^

Standardised tACS will be applied with a frequency of 10 Hz, which is in the alpha frequency and is consistent with other alpha-tACS studies.^47^ The tACS electrodes will be positioned over AFz and Oz, according to the 10–10 system for electrode placement (figure 2). This electrode placement results in the generation of electrical fields in brain areas that demonstrate altered connectivity in delirium, including the dorsolateral prefrontal cortex, precuneus and posterior cingulate cortex.^22 23^ At the beginning of stimulation, the intensity will ramp up for 30 s to 2.0 mA peak to peak, while at the end of stimulation, the intensity will ramp down for 30 s to 0 mA.

#### Personalised tACS

For personalised tACS, settings will be based on a computational model for delirium and a virtual trial. To achieve this, this study will use a computational model capable of mimicking in silico the EEG findings that have been observed in delirium. A network of neural masses with each neural mass (ie, the smallest subsection of the network) representing a population of excitatory and inhibitory neurons in the brain will be used. By modifying the excitatory-inhibitory balance and/or subcortical input to the neural masses, different pathologies can be simulated. The model generates multiple channel EEG-like output, allowing for quantitative analysis of outcomes of different model parameters. Model parameters will be manipulated to simulate neuronal/synaptic changes during delirium as well as individual (personalised) brain activity and functional connectivity, resulting in EEG characteristics that are similar to that observed in a particular patient, amounting to a personalised disease model. Thereafter, the effect of various tACS parameters will be simulated to counter delirium mechanisms. These strategies will differ with regard to the electrode location and stimulation frequency. The different quantitative measures resulting from the model will be analysed similarly to patient EEG data, predicting which electrode placement and stimulation frequency will result in the most optimal treatment response regarding power spectrum and connectivity characteristics. In this context, optimal treatment response is defined as a change of spectral and connectivity characteristics of the model output in the direction of a healthy state. The optimal, individualised tACS protocol will thereafter be applied as personalised delirium treatment. Settings will be determined once during the first session and will remain unchanged in remaining sessions. All patients in the active treatment arm will receive tACS with an intensity of 2.0 mA (peak to peak) and a stimulation duration of 30 min.

We are currently investigating the optimal way to fit a network of neural masses to an individual patient with delirium, allowing performance of a virtual trial with the specified outcome parameters. In this phase, several strategies will be considered: a disease model tailored at multiple dimensions to the individual neurophysiology,^48^ a model tailored to the individual peak frequency^49^ or spatial modelling of individual brain activity. The results of this development process will be published in a separate paper describing the details of this approach and the most effective strategy will be used in the second phase of the trial.

#### Sham stimulation

The procedure for sham stimulation will be identical to either standardised or personalised tACS, except for the electrical current administered. After the 5-digit pin code is entered, which enables sham stimulation, the tACS device will ramp up to 2.0 mA peak to peak for 30 s, stimulate for 60 s and ramp down for 30 s to 0 mA. This mimics the perception of actual tACS stimulation and improves blinding. To evaluate the effectiveness of blinding, both the participant and the researcher will be asked to guess the group allocation after the first and last treatment session (online supplemental appendix 1).

### Outcomes

#### Pilot study outcomes

During the pilot phase, data regarding the percentage of fully completed tACS sessions will be recorded as well as increased care requirements within 1 hour following tACS administration. An increase in care requirement is defined as a (medication-based) intervention (eg, for heightened agitation or skin issues resulting from the electrodes), fixation, or transfer to unit with more advanced care (eg, the intensive care unit). Furthermore, duration of delirium will be recorded as defined in the secondary outcomes below. On analysis of these findings, adjustments to the protocol may be proposed and will be submitted to the Medical Research Ethics Committee (MREC) for approval before the start of the main study phase, if deemed necessary.

#### Main study primary outcome

##### Relative delta power

An 18-min resting state EEG recording will be conducted by a trained clinical researcher directly before and after the first tACS session. EEG recordings will be obtained using a 64-channel Biosemi ActiveTwo EEG system with active gel electrodes (Biosemi B.V., Amsterdam, Netherlands) at a sampling rate of 2048 Hz. Active electrodes, wherein each electrode has its own amplifier, are employed to reduce artefacts due to enhanced signal-to-noise ratio. EEG data will be visually inspected for eye movement and muscle artefacts. A minimum of 80 s of eyes closed artefact-free data will be analysed. Data will undergo FIR bandpass filtering in the following frequency bands: delta (0.5–4 Hz), theta (4–8 Hz), alpha (8–13 Hz), low beta (13–20 Hz) and high beta (20–30 Hz). Relative delta power will be calculated by dividing the total power within the delta frequency band (0.5–4 Hz) by the total power across frequency bands from 0.5 to 20 Hz. The upper limit of the frequency band is limited to 20 Hz to reduce the impact of muscle artefacts and high-frequency noise on the relative delta power calculation.^50^

##### Secondary outcomes

- Delirium duration assessed by the number of days with delirium during the treatment period (up to 14 days). A delirium-positive day is defined as having an Intensive Care Delirium Screening Checklist (ICDSC)^51^ score of ≥4. A score of –4 or lower on the RASS followed by an ICDSC score ≥4 is counted as a delirium day. For days where ICDSC is missing (eg, due to limited staff availability on some weekends), days with a Delirium Observation Screening Scale (DOSS)^52^ score ≥3 will also count as a delirium day. The DOSS is administered as standard of care.
- Delirium severity as assessed by the cumulative ICDSC score per participant recorded on days with delirium during the treatment period. In instances where ICDSC scores are unavailable, scores will be estimated using information from the electronic patient record.
- Quantitative EEG measures include peak frequency, spectral analysis and connectivity measures such as the phase lag index,^53^ corrected amplitude envelope correlation^48^ and topological measures based on the minimum spanning tree.^54 55^
- Length of hospital stay as assessed by the total number of days admitted to the hospital.
- Cognitive status 3 months after the first tACS session as assessed by the TICS-M.^43 44^
- Presence and duration of sensations related to tACS treatment including tingling sensations, itching, mild transient redness of the skin and discomfort on the region of stimulation with the sensation questionnaire developed for this study (online supplemental appendix 1).
- The treatment burden, perception of receiving either sham or active tACS and patients’ perceptions of the therapeutic relationship with the researcher(s) will be evaluated using the questionnaires on feasibility, or blinding and subjective treatment experience, which have been developed for this study (online supplemental appendix 1).

### Safety reporting

#### Adverse events

AEs are defined as any undesirable experience occurring to a participant during the study, whether or not considered related to the experimental intervention. Given that hospitalised patients often experience AEs, only potential study-related AEs reported by the participant or observed by the study team during the timeframe of tACS treatment will be documented in the case report form. These include sensations related to tACS (ie, itch, pain, burn, heat, iron taste, headache, neck pain, phosphenes, dizziness and nausea), behaviour suggesting of increase in delirium severity such as increase in use of antipsychotics, patient fixation, falling out of bed and self-removal of a line, tube or drain, and a possible epileptic seizure. On each treatment day, the study team will screen the electronic patient record and consult with the treating physician or nurse about any health changes since the previous tACS session. Any event potentially related to the study procedures will be classified as an AE.

#### Serious adverse events (SAEs)

An SAE is any untoward medical occurrence or effect that

- results in death;
- is life threatening (at the time of the event);
- requires hospitalisation or prolongation of existing inpatients’ hospitalisation;
- results in persistent or significant disability or incapacity;
- any other important medical event that did not result in any of the outcomes listed above due to medical or surgical intervention but could have been based on appropriate judgement by the investigator.

For the purpose of this study, an SAE is defined according to the definition above, within the timeframe of tACS treatment, which includes up to 24 hours after the last tACS session. It should be noted that infectious diseases such as pneumonia, wound infection, sepsis, (postoperative) haemorrhage, or laboratory disturbances, such as hyponatraemia or hypokalaemia that may prolong inpatients’ hospitalisation or may be life threatening, will not be considered as an SAE. This exclusion is due to the frequency of these complications in the population being studied, which is unrelated to tACS treatment.

### Statistical analysis

For the analysis of the primary study parameter, a per-protocol analysis will be used. The sole criterion for inclusion in the analysis is that a participant has completed the initial tACS session and EEG recordings. Changes in relative delta power will be assessed using separate linear mixed models for standardised and personalised tACS compared with sham, with relative delta power as the dependent variable, time*group and study centre as fixed factors, and participant as a random factor. Data analysis will be performed blinded for treatment allocation. A significance level of p=0.05 (two-tailed) will be applied. To retain sensitivity to detect potential effects in this novel area of research, no adjustment for multiple comparisons will be made. In cases of deviations from the linear mixed model, robust models and non-parametric alternatives will be considered. Subgroup analysis will be conducted by including additional fixed factors to the mixed models, such as delirium aetiology, sex and age. Functional outcomes, along with other quantitative EEG measurements and cognitive outcomes, will be analysed using non-parametric or parametric tests depending on the distribution of scaled test results. Blinding success for participants as well as researchers will be tested using a χ^2^ test.

### Interim analysis

Preplanned interim analyses will be conducted after the pilot study to assess the percentage of fully completed tACS sessions, increased care requirements within 1 hour following tACS administration, and differences in delirium duration between the active and sham tACS treatment groups. For these analyses, a Student’s t-test will be employed if data follow a normal distribution, whereas a Mann-Whitney test will be used for skewed distributions. Results will be shared with the MREC before proceeding with the main study phase.

### Patient and public involvement

Patients and/or the public were not involved in the design, or conduct, or reporting, or dissemination plans of this study.

### Data management, monitoring and access

The handling of personal data will adhere to the EU General Data Protection Regulation and the Dutch Act on Implementation of the General Data Protection Regulation. Study data will be collected and managed using Castor EDC, a secure electronic case record form (eCRF) accessible via the internet. Investigators will be assigned personal usernames and passwords, and all data transfers will be encrypted. Only data essential to addressing the research question outlined in this protocol will be collected and stored. All data will be pseudonymised and treated confidentially. Only necessary study members will have access to this subject identification list. Investigators will electronically sign to confirm that eCRF entries are accurate and complete. Source documents will be securely stored in a locked filing cabinet, accessible only to authorised research personnel, and archived for the legally mandated period. Before the start of the study, it is agreed which documents serve as source data for eCRF. Monitoring will be conducted in accordance with national laws and International Conference on Harmonisation-Good Clinical Practice (ICH-GCP) guidelines. Given the low-risk intervention, there will not be an independent data monitoring committee.

## Ethics and dissemination

The study has been approved by the MREC of the Utrecht UMC (23-198) and the Institutional Review Boards of participating centres. This study will be conducted according to the principles of the Declaration of Helsinki (see for the most recent version: www.wma.net) and in accordance with the Medical Research Involving Human Subjects Act (WMO) and other guidelines, regulations and acts. All substantial amendments will be notified to the local MREC. The trial results will be made accessible to the public in a peer-reviewed journal, preferably open access.

### Trial status

Protocol version 1.5, June 2024. The trial is currently in the recruitment phase. Initial approval of the MREC was granted in January 2024. The first participant was included in April 2024. The expected end date for the trial is April 2027.

## supplementary material

10.1136/bmjopen-2024-092165 online supplemental appendix 1

10.1136/bmjopen-2024-092165 online supplemental appendix 2
