# Supplementary material for: DELirium treatment with Transcranial Electrical Stimulation (DELTES): study protocol for a multicentre, randomised, double-blind, sham-controlled trial
Source: BMJ Open. 2024 Nov 2;14(11):e092165. doi: 10.1136/bmjopen-2024-092165 (PMC11535714; doi:10.1136/bmjopen-2024-092165)
Supplement: online supplemental appendix 1 [file bmjopen-14-11-s001.docx]

# Appendix 1

# Sensation questionnaire

*Did the patient perceive anything unusual during the stimulation? Use the scale below to answer the following questions regarding different sensations and the extent to which the patient felt them.*

**1. Itching** none mild severe no reliable answer

**2. Pain** none mild severe no reliable answer

**3. Burning sensation**

none mild severe no reliable answer

**4. Heat sensation under electrodes** none mild severe no reliable answer

**5. Iron taste** none mild severe no reliable answer

**6. Headache** none mild severe no reliable answer

**7. Neck pain** none mild severe no reliable answer

**8. Phosphenes** none mild severe no reliable answer

**9. Dizziness** none mild severe no reliable answer

**10. Nausea** none mild severe no reliable answer

| *Only ask the following questions if sensations were perceived in questions 1 to 10.*  **11. When did the sensations begin?**  at the beginning of the stimulation (the first few minutes)   in the middle of the stimulation   at the end of the stimulation (the last few minutes) |
| --- |
| **12. How long did the sensations last?**  less than a minute   several minutes   longer than 5 minutes  (almost) the entire stimulation |

| *The following questions are directed to the researcher.*  **13. To what extent did you observe pain or discomfort in the patient during the stimulation?**  none mild severe no reliable answer |
| --- |
| *Only ask questions 14 and 15 if pain or discomfort was observed in question 13.*  **14. When did you notice the pain or discomfort in the patient?**  at the beginning of the stimulation (the first few minutes)   in the middle of the stimulation   at the end of the stimulation (the last few minutes) |
| **15. How long did you observe the pain or discomfort in the patient?**  less than a minute   several minutes   longer than 5 minutes  (almost) the entire stimulation |

# Blinding and subjective treatment experience questionnaire

**Do you think you received real or sham (placebo) stimulation?**

real placebo (sham) don’t know

How certain are you of your choice? (0% = not at all certain, 100% = absolutely certain) _______ %

**How did you experience the contact with the researcher(s)?**

excellent

good

neutral

poor

very poor

don’t

**To be filled out by the researcher:
Do you think the patient received real or placebo stimulation?**

placebo (sham)

real

**Experiences with tACS Treatment for Delirium**

**Version for patients**

**You have just undergone treatment with brain stimulation (tACS). We would like to hear from you about how you experienced this treatment and therefore ask you to answer the questions below. The answers will help us improve the treatment.**

What did you think of the treatment?

________________________________________________________________________________________________________________________________________________________________________________________________________________________________________________________________________________________________________________________________________________________________________

How burdensome did you find this treatment?

| Very much (1) | A little (2) | Neutral (3) | Not (4) | Not at all (5) |
| --- | --- | --- | --- | --- |
|  |  |  |  |  |

How would you rate the feasibility of this treatment? Please rate between 1 (poor) and 10 (excellent)

**Experiences with tACS Treatment for Delirium**

**Version for relatives**

**Your relative has just undergone treatment with brain stimulation (tACS). We would like to hear from you about how you experienced this treatment for your relative and therefore ask you to answer the questions below. The answers will help us improve the treatment.**

What is your relationship to the patient? Circle what applies:

Spouse / partner / son / daughter / parent / guardian / conservator / mentor / other, namely __________________________________

What did you think of the treatment for your relative?

________________________________________________________________________________________________________________________________________________________________________________________________________________________________________________________________________________________________________________________________________________________________________

How burdensome did you find this treatment for your relative?

| Very much (1) | A little (2) | Neutral (3) | Not (4) | Not at all (5) |
| --- | --- | --- | --- | --- |
|  |  |  |  |  |

How would you rate the feasibility of this treatment? Please rate between 1 (poor) and 10 (excellent)

**Experiences with tACS Treatment for Delirium**

**Version for health care provider**

**One of your patients has just undergone treatment with brain stimulation (tACS). We would like to hear from you about how you experienced this treatment for the patient and therefore ask you to answer the questions below. The answers will help us improve the treatment.**

Which healthcare provider filled out this questionnaire? Circle what applies:

Nurse / Resident physician / Specialist

What did you think of the treatment for the patient?

________________________________________________________________________________________________________________________________________________________________________________________________________________________________________________________________________________________________________________________________________________________________________

How burdensome did you find this treatment for the patient?

| Very much (1) | A little (2) | Neutral (3) | Not (4) | Not at all (5) |
| --- | --- | --- | --- | --- |
|  |  |  |  |  |

How would you rate the feasibility of this treatment? Please rate between 1 (poor) and 10 (excellent)
