# Supplementary material for: DELirium treatment with Transcranial Electrical Stimulation (DELTES): study protocol for a multicentre, randomised, double-blind, sham-controlled trial
Source: BMJ Open. 2024 Nov 2;14(11):e092165. doi: 10.1136/bmjopen-2024-092165 (PMC11535714; doi:10.1136/bmjopen-2024-092165)
Supplement: online supplemental appendix 2 [file bmjopen-14-11-s002.docx]

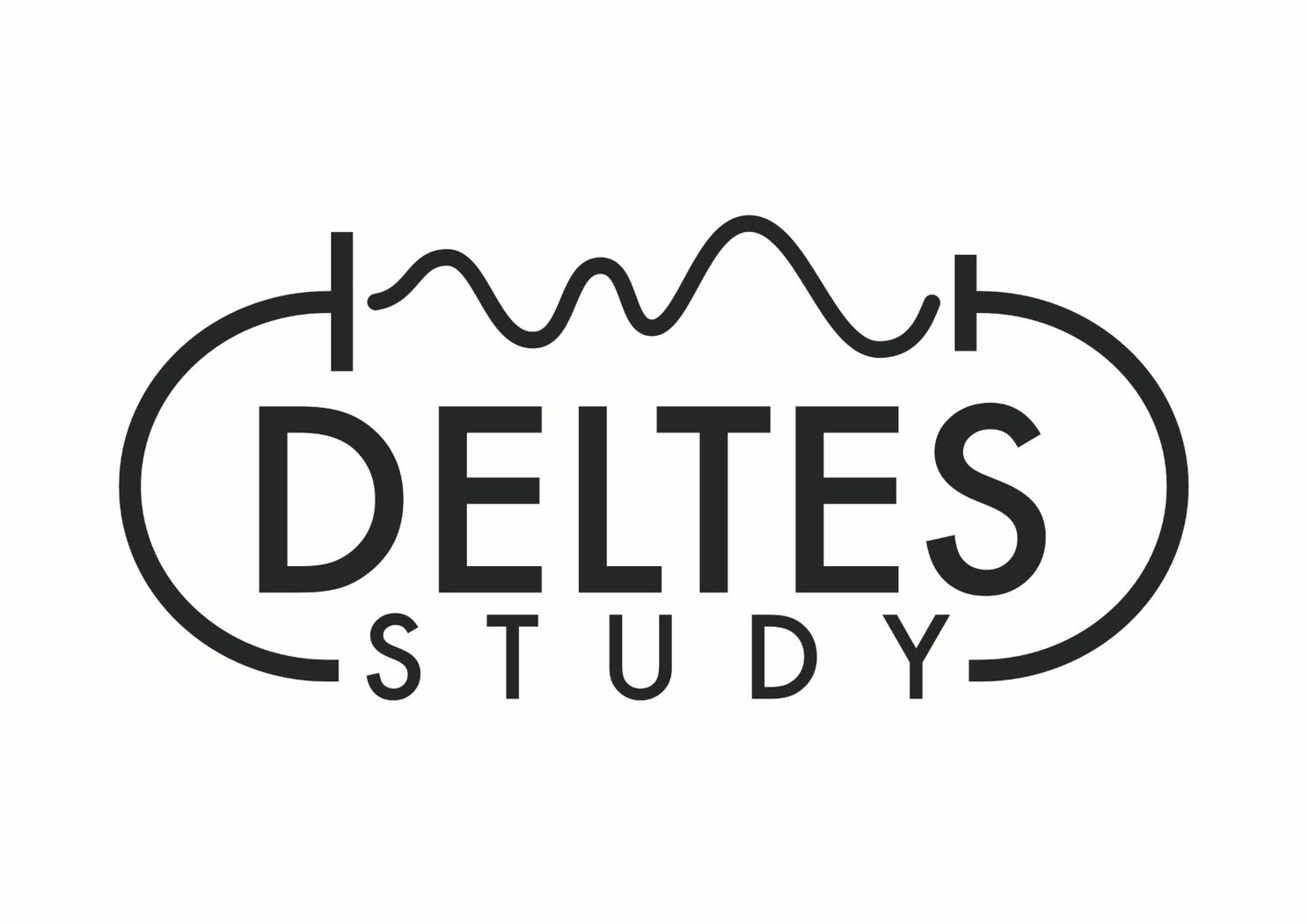


**Delirium treatment with Transcranial Electrical Stimulation**

Een studie naar het effect van elektrische hersenstimulatie bij mensen met delirium (acute verwardheid)

**Informatiebrief voor wettelijk vertegenwoordiger**

**inclusief toestemmingsformulier**

Versie 2.3, 01 mei 2024

Dossiernummer NL 84043.041.23

Hoofdaanvrager: UMC Utrecht

Onder leiding van Prof. dr. A.J.C. Slooter

Geachte heer/mevrouw,

U ontvangt deze brief omdat een naaste van u is opgenomen in het ziekenhuis en momenteel acute verwardheid (ook wel delier of delirium genoemd) heeft. Wij vragen u als wettelijke vertegenwoordiger om toestemming voor deelname van uw naaste aan een medisch-wetenschappelijk onderzoek. Uw naaste is op dit moment verward, en daarom is het voor hem of haar niet mogelijk om zelf een beslissing te nemen. Zodra dit weer kan, zullen wij uw naaste zelf ook om toestemming vragen voor deelname aan het onderzoek en het gebruiken van de verzamelde gegevens.

U leest hier om wat voor onderzoek het gaat, wat het voor uw naaste betekent, en wat de voordelen en nadelen zijn. Het is veel informatie. Wilt u de informatie doorlezen en beslissen of u toestemming geeft voor deelname aan het onderzoek door uw naaste? Als u toestemming wilt geven, kunt u het formulier invullen dat u vindt in bijlage C.

**Stel uw vragen**

U kunt uw beslissing nemen met de informatie die u in deze informatiebrief vindt. Daarnaast raden we u aan om dit te doen:

- Stel vragen aan de onderzoeker die u deze informatie geeft.

- Praat met uw partner, familie of vrienden over dit onderzoek.

- Stel vragen aan de onafhankelijk deskundige. Voor contactgegevens zie bijlage A

- Lees de informatie op [www.rijksoverheid.nl/mensenonderzoek](http://www.rijksoverheid.nl/mensenonderzoek).

1. **Algemene informatie**

Dit onderzoek is opgezet door het UMC Utrecht in samenwerking met het Radboudumc en het HagaZiekenhuis. Hieronder noemen we het UMC Utrecht steeds de ‘opdrachtgever’. Onderzoekers, dit kunnen ook artsen of onderzoeksverpleegkundigen zijn, voeren het onderzoek uit in het UMC Utrecht, Radboudumc en het HagaZiekenhuis. Deelnemers aan een medisch-wetenschappelijk onderzoek worden vaak proefpersonen genoemd. Zowel patiënten als mensen die gezond zijn, kunnen proefpersoon zijn. Aan dit onderzoek zullen 159 proefpersonen deelnemen. De medisch-ethische toetsingscommissie NedMec heeft dit onderzoek goedgekeurd.

1. **Wat is het doel van het onderzoek?**

In dit onderzoek bekijken we of stimulatie van de hersenen met een zwak elektrisch stroompje (transcraniële elektrische stimulatie of tACS) werkt als nieuwe behandeling voor delier (ook wel delirium). We vergelijken de werking van de behandeling met de werking van een niet-werkzame controlebehandeling (placebo).

1. **Wat is de achtergrond van het onderzoek?**

Tijdens een ziekenhuisopname kan iemand opeens in de war raken; dit wordt delier of delirium genoemd. Een delier kan bijvoorbeeld door een operatie of ontsteking ontstaan. Deze verwardheid is meestal tijdelijk en duurt vaak enkele uren tot dagen, en soms zelfs weken. Een delier kan ernstige gevolgen hebben voor de patiënt: bijvoorbeeld langer verblijf in het ziekenhuis, langer aan de beademing of blijvende vergeetachtigheid. Daarom zijn we op zoek naar een nieuwe, effectieve behandeling voor delier.

Als je bij iemand met een delier hersengolven meet met sensoren op het hoofd (EEG), zien we dat de hersengolven zijn vertraagd. Een nieuwe behandeling die hier mogelijk iets aan kan doen is elektrische hersenstimulatie, of tACS. We weten namelijk dat deze behandeling veilig is en de hersengolven kan versnellen, alleen is dit niet bij patiënten met een delier onderzocht. Bij een deel van de patiënten doen we een standaard tACS-behandeling. Bij een ander deel kijken we of we de instellingen kunnen aanpassen op de hersengolven die we meten bij de patiënt. Hierdoor is de behandeling voor iedere persoon zo passend mogelijk (gepersonaliseerd).

1. **Voorbereiding van het onderzoek**

We willen eerst weten of uw naaste geschikt is om mee te doen. Daarom bekijkt de onderzoeker:

- Of een delier inderdaad aanwezig is met behulp van een vragenlijst.
- Of er redenen zijn om geen elektrische stimulatie te kunnen krijgen zoals een pacemaker, aandoeningen aan de huid of epilepsie.
- Of er andere redenen zijn om niet mee te doen aan het onderzoek zoals coma, dementie, doofheid of blindheid.

Als u toestemming geeft voor deelname van uw naaste komen we dagelijks langs voor een behandeling tijdens de ziekenhuisopname, tot maximaal 14 dagen. Drie maanden later volgt nog een telefoongesprek van ongeveer 15 minuten met een paar testjes van het geheugen.

De elektrische stimulatie kan op verschillende manieren worden ingesteld. We onderzoeken daarom of er een verschil is tussen elektrische stimulatie met standaardinstellingen, of gepersonaliseerde instellingen. Loting bepaalt of uw naaste de standaard of gepersonaliseerde behandeling krijgt. Wanneer uw naaste de gepersonaliseerde behandeling krijgt, zal de elektrische stimulatie afgestemd zijn op de hersengolven van de patiënt. Hiervoor krijgt uw naaste voorafgaand aan de behandeling een extra EEG.

Om te kunnen onderzoeken of de elektrische stimulatie (tACS) effect heeft, zal niet iedereen de actieve stimulatie krijgen. Een deel van de mensen zal elektrische stimulatie (tACS) krijgen. Een ander deel van de mensen krijgt een niet-werkzame controlestimulatie. Loting bepaalt welke behandeling uw naaste krijgt, oftewel of uw naaste de actieve of niet-werkzame controlestimulatie krijgt. U, uw naaste en de onderzoeker weten niet of uw naaste actieve of niet-werkzame controlestimulatie krijgt. Als het voor de gezondheid van uw naaste belangrijk is, kan dit wel worden opgezocht.

1. **Hoe verloopt het onderzoek?**

Wanneer u toestemming geeft voor deelname van uw naaste verzamelen we een aantal basisgegevens uit het medisch dossier, zoals leeftijd, geslacht, medische voorgeschiedenis, welke behandeling uw naaste eerder tijdens de opname heeft ondergaan of momenteel ondergaat en welke medicatie uw naaste ten tijde van het onderzoek krijgt toegediend. Daarna beginnen we met een korte vragenlijst om de kenmerken van delier te meten. Wanneer uw naaste de gepersonaliseerde behandeling krijgt, zal de elektrische stimulatie afgestemd zijn op de hersengolven. Hiervoor krijgt uw naaste voorafgaand aan de behandeling een extra EEG, dit duurt ongeveer 40 minuten. Later op de dag krijgt uw naaste de eerste stimulatie. Direct voor en na de stimulatie wordt ook een EEG gemaakt om het effect van de behandeling te meten. Samen met de stimulatie duurt dit ongeveer 95 minuten.

Zolang uw naaste is opgenomen in het ziekenhuis en een delier heeft, komen we dagelijks langs voor een stimulatie, tot maximaal 14 dagen. Ook wordt er dan een korte vragenlijst afgenomen over de kenmerken van delier. In totaal zijn we ongeveer 45 minuten per dag aanwezig, maar tijdens de stimulatie (30 minuten) hoeft uw naaste niks te doen.

Wanneer uw naaste wordt ontslagen uit het ziekenhuis of het delier verdwenen is, willen we graag nog één keer een EEG maken. Daarnaast zouden we tijdens deze afspraak enkele vragenlijsten willen afnemen over de kenmerken van delier en ervaringen met de behandeling (ongeveer 45 minuten in totaal).

Tot slot willen we uw naaste, ongeveer 3 maanden na de startdatum van het onderzoek, één keer bellen voor een paar vragen over het geheugen. Deze telefonische afspraak duurt ongeveer 15 minuten.

*Wat is er anders dan bij gewone zorg?*

Tijdens het onderzoek krijgt uw naaste ook de normale behandeling van delier zoals medicijnen, het aanhouden van een dag-nachtritme en oorzaken zoals ontsteking behandelen (indien van toepassing). Hier bovenop komt de elektrische stimulatie. Mochten er andere behandelingen of onderzoeken moeten plaatsvinden, dan krijgen die voorrang en kunnen we de studiehandelingen bijvoorbeeld een dag overslaan.

1. **Wat wordt er van uw naaste verwacht?**

We willen graag dat het onderzoek goed verloopt. Daarom kan uw naaste niet ook nog aan een ander medisch-wetenschappelijk onderzoek naar delier meedoen.

Het is belangrijk dat u contact opneemt met de onderzoeker:

- als u besluit uw toestemming voor deelname aan het onderzoek in te trekken.
- als uw contactgegevens wijzigen.

1. **Van welke bijwerkingen, nadelige effecten of ongemakken kan uw naaste last krijgen?**

*Het meten van hersengolven met EEG*

Een EEG is een middel om hersenactiviteit (hersengolven) te meten in het brein via elektroden. Uw naaste krijgt een soort badmuts met elektroden op. Het aansluiten van een EEG duurt ongeveer 10 minuten. Vervolgens zal uw naaste gevraagd worden om 15 minuten de ogen gesloten te houden en stil te liggen.

Aan een EEG zijn geen risico’s of bijverschijnselen verbonden. Een EEG doet geen pijn.


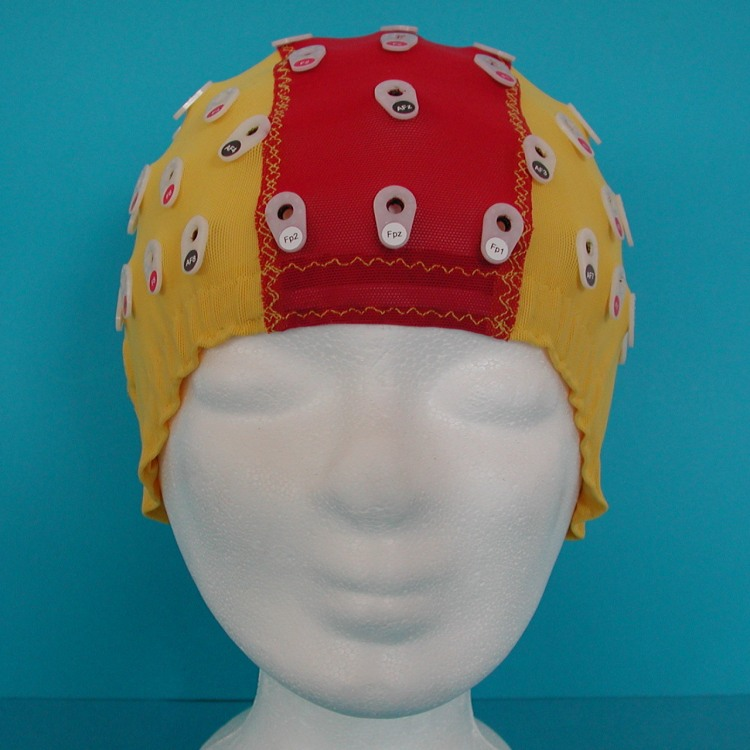


**Afbeelding 1**. De EEG-muts die wordt opgezet om de hersengolven te meten.

*De behandeling met tACS (elektrische stimulatie)*

Bij een behandeling met tACS (elektrische stimulatie) worden er twee kleine vochtige elektroden (dunne sponsachtige lapjes met een draadje eraan) op het hoofd geplaatst waarna een persoon dagelijks een halfuur een klein stroompje krijgt. Het apparaat dat in dit onderzoek wordt gebruikt voor tACS is de BrainBox Nurostym. Dit apparaat is gekeurd volgens de normen van de Europese Unie voor gebruik bij neuropsychiatrische aandoeningen (CE-markering).

Van deze behandeling is bekend dat het in sommige gevallen lichte bijwerkingen kan geven:

- Tijdelijk een gevoel van jeuk, branderigheid of warmte op de huid bij de elektroden. Dit trekt vanzelf weer weg.
-
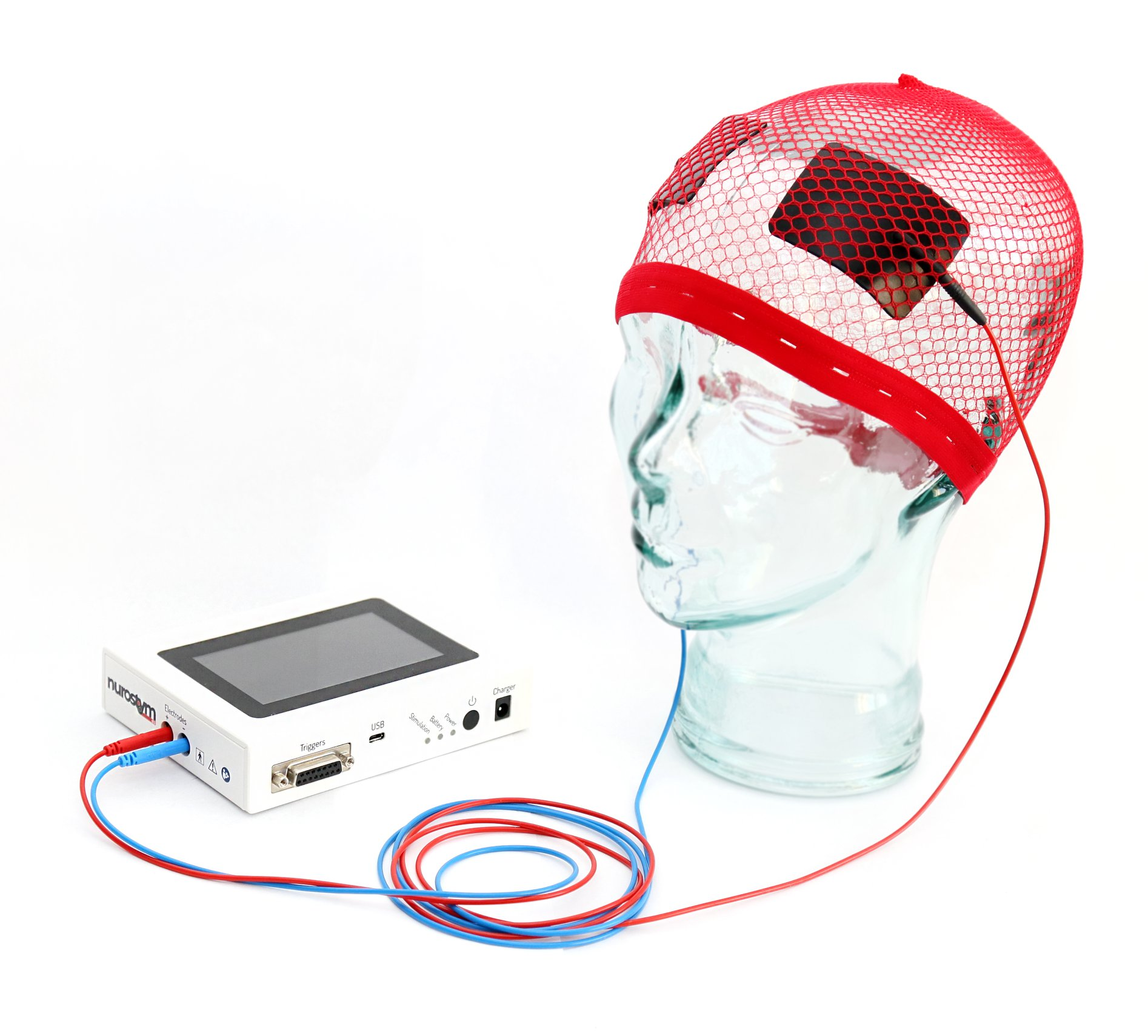
Hoofdpijn, moeheid of moeite met concentreren na de behandeling, die vaak binnen enkele uren weer wegtrekt.

De volgende bijwerkingen komen zeer weinig voor:

- Beschadiging (brandwondje) van de huid bij de elektroden. Deze bijwerking proberen we te voorkomen door de elektroden goed contact te laten maken met de huid.

tACS (elektrische stimulatie) wordt bij verschillende patiëntengroepen veilig gebruikt in onderzoeksverband, maar het is de eerste keer dat er onderzoek wordt gedaan naar de werking van tACS bij mensen met een delier. Daarom kunnen er ook bijwerkingen optreden die we nu nog niet weten.

**Afbeelding 2.** De behandeling met tACS.

1. **Deelname van wilsonbekwame proefpersonen**

Het kan gebeuren dat degene die u vertegenwoordigt zich op een bepaald moment tijdens het onderzoek verzet (niet meewerkt). De onderzoeker moet het onderzoek dan direct stoppen. Het is moeilijk om precies te omschrijven wat verzet is. Voor de start van het onderzoek overleggen we met u wat wij zien als verzet. De onderzoeker zal zich houden aan de Gedragscode verzet bij wilsonbekwame (psycho)geriatrische patiënten in het kader van de Wet Medisch-Wetenschappelijk Onderzoek met mensen.

1. **Wat zijn de voordelen en de nadelen als uw naaste meedoet aan het onderzoek?**

Mogelijke voordelen zijn:

- De behandeling met tACS kan er mogelijk aan bijdragen dat het delier het korter duurt of minder ernstig is, maar dit is niet zeker. Omdat we nu voor het eerst tACS als behandeling voor delier onderzoeken is dit niet bekend.
- Deelname aan het onderzoek kan bijdragen aan meer kennis over de aandoening.

Mogelijke nadelen zijn:

- Uw naaste kan bijwerkingen of nadelige effecten van tACS ervaren.
- Uw naaste kan last hebben van de metingen tijdens het onderzoek.
- Meedoen aan het onderzoek kost uw naaste extra tijd.

U beslist of uw naaste meedoet aan het onderzoek. Deelname is vrijwillig. Als u geen toestemming geeft voor deelname, wordt uw naaste op de gebruikelijke manier behandeld voor zijn of haar aandoening. U kunt uw toestemming op elk moment intrekken. U hoeft niet te zeggen waarom u uw toestemming intrekt. Wel moet u dit direct melden aan de onderzoeker. De gegevens die tot dat moment zijn verzameld, worden gebruikt voor het onderzoek.

1. **Wanneer stopt het onderzoek?**

In deze situaties stopt voor het onderzoek voor uw naaste:

- Het einde van het onderzoek is bereikt.
- U kiest om uw toestemming in te trekken. U hoeft er niet bij te vertellen waarom.
- U, de behandelaar(s) van uw naaste of de onderzoeker(s) zien aanhoudende tekenen van verzet bij uw naaste. Tekenen van verzet kunnen verbale (spraak) of non-verbale (lichamelijke) uitingen zijn en bestaat vaak uit afwerend gedrag.
- Het UMC Utrecht, Radboudumc, HagaZiekenhuis, de overheid of de beoordelende medisch-ethische toetsingscommissie besluit om het onderzoek te stoppen.

Het gehele onderzoek is afgelopen als alle deelnemers klaar zijn.

1. **Wat doen we met de gegevens van uw naaste?**

Voor dit onderzoek worden de persoonsgegevens van uw naaste verzameld, gebruikt en bewaard. Het gaat om gegevens zoals naam, geslacht, contactgegevens, adres, geboortedatum en om gegevens over de gezondheid van uw naaste. Het verzamelen, gebruiken en bewaren van deze gegevens is nodig om de vragen die in dit onderzoek worden gesteld te kunnen beantwoorden en de resultaten te kunnen publiceren.

*Hoe beschermen we de privacy?*

Om de privacy van uw naaste te beschermen geven wij de gegevens van uw naaste een code. Op al deze gegevens zetten we alleen deze code. De sleutel van de code bewaren we op een beveiligde plek in het ziekenhuis. Als we de gegevens van uw naaste verwerken, gebruiken we steeds alleen die code. Ook in rapporten en publicaties over het onderzoek kan niemand terughalen dat het over uw naaste ging.

*Wie kunnen de gegevens van uw naaste zien?*

Sommige personen kunnen wel de naam en andere persoonlijke gegevens van uw naaste zonder code inzien. Dit kunnen gegevens zijn die speciaal voor dit onderzoek zijn verzameld, maar ook gegevens uit het medisch dossier. Dit zijn mensen die controleren of de onderzoekers het onderzoek goed en betrouwbaar uitvoeren. Deze personen kunnen bij de gegevens van uw naaste komen:

- Leden van de commissie die de veiligheid van het onderzoek in de gaten houdt.
- Een controleur die door het UMC Utrecht is ingehuurd.
- Nationale toezichthoudende autoriteiten.

Deze personen houden de gegevens van uw naaste geheim. Voor inzage door deze personen vragen wij u toestemming te geven. De Inspectie Gezondheidszorg en Jeugd kan zonder uw toestemming de gegevens van uw naaste inzien.

*Hoelang bewaren we de gegevens van uw naaste?*

Alle studiegegevens worden in een beveiligde omgeving bewaard tot 15 jaar na het einde van het onderzoek. Daarna worden de gegevens van uw naaste vernietigd.

*Mogen we de gegevens van uw naaste gebruiken voor ander onderzoek?*

De gegevens van uw naaste kunnen erg waardevol zijn voor ander wetenschappelijk onderzoek op het gebied van delier of de behandeling met tACS, zowel binnen als buiten de EU. Buiten de EU gelden mogelijk andere regels voor bescherming van persoonsgegevens. Daarom vragen wij uw toestemming voor het doorsturen van de gegevens. De gegevens worden alleen gecodeerd doorgegeven en zijn niet naar uw naaste herleidbaar. Geeft u geen toestemming voor het delen van de gegevens van uw naaste? Dan kan uw naaste nog steeds meedoen aan dit onderzoek. Uw naaste krijgt dan dezelfde zorg.

*Wat gebeurt er bij onverwachte ontdekkingen?*

Tijdens het onderzoek kunnen we toevallig iets vinden dat niet direct van belang is voor het onderzoek maar wel voor de gezondheid van uw naaste. De onderzoeker neemt dan contact op met de specialist van uw naaste. De specialist bespreekt met u en uw naaste wat er moet gebeuren. De kosten hiervan vallen onder de zorgverzekering van uw naaste. U geeft met het formulier toestemming voor het informeren van de specialist van uw naaste.

*Kunt u of uw naaste de toestemming voor het gebruik van uw gegevens weer intrekken?*

U kunt uw toestemming voor het gebruik van de gegevens van uw naaste op ieder moment intrekken. Zeg dat dan tegen de onderzoeker. Maar let op: de onderzoeksgegevens die zijn verzameld tot het moment dat u uw toestemming intrekt worden nog wel gebruikt in het onderzoek. Wanneer uw naaste na het verdwijnen van het delier zijn of haar toestemming intrekt, kan hij of zij ervoor kiezen alle verzamelde gegevens te laten verwijderen.

*Wilt u meer weten over de privacy van de gegevens?*

- Wilt u meer weten over de rechten bij de verwerking van persoonsgegevens van uw naaste? Kijk dan op [www.autoriteitpersoonsgegevens.nl](http://www.autoriteitpersoonsgegevens.nl).
- Heeft u als naaste vragen over uw rechten? Of heeft u een klacht over de verwerking van de persoonsgegevens van uw naaste? Neem dan contact op met degene die verantwoordelijk is voor de verwerking van uw persoonsgegevens. Voor dit onderzoek is dat:
  - Zie bijlage A voor contactgegevens.
- Als u klachten heeft over de verwerking van de persoonsgegevens van uw naaste, raden we u aan om deze eerst te bespreken met het onderzoeksteam. U kunt ook naar de Functionaris Gegevensbescherming van het ziekenhuis waar uw naaste wordt behandeld gaan. Of u dient een klacht in bij de Autoriteit Persoonsgegevens.

1. **Krijgt uw naaste een vergoeding als hij of zij meedoet aan het onderzoek?**

De onderzoeken, extra testen en behandeling voor het onderzoek kosten u en uw naaste niets. U en/of uw naaste wordt niet betaald voor het meedoen aan dit onderzoek.

1. **Is uw naaste verzekerd tijdens het onderzoek?**

Voor iedereen die meedoet aan dit onderzoek is een verzekering afgesloten via het UMC Utrecht. De verzekering dekt schade door het onderzoek. In bijlage B vindt u meer informatie over de verzekering en de uitzonderingen. Daar staat ook aan wie u schade kunt melden.

1. **Heeft u vragen?**

Vragen over het onderzoek kunt u stellen aan de onderzoeker. Wilt u advies van iemand die er geen belang bij heeft? Ga dan naar de onafhankelijk deskundige, voor contactgegevens zie bijlage A. Hij weet veel over het onderzoek, maar werkt niet mee aan dit onderzoek. Heeft u een klacht? Bespreek dit dan met de onderzoeker of de behandelend arts. Wilt u dit liever niet? Ga dan naar de klachtenfunctionaris van uw ziekenhuis. In bijlage A staat waar u die kunt vinden.

U kunt uw beslissing nemen met de informatie die u in deze informatiebrief vindt. Daarnaast raden we u aan om al uw vragen te stellen aan de onderzoekers, over het onderzoek te praten met naasten en de informatie te lezen op [www.rijksoverheid.nl/mensenonderzoek](http://www.rijksoverheid.nl/mensenonderzoek).

1. **Hoe geeft u toestemming voor het onderzoek?**

Wanneer u voldoende bedenktijd heeft gehad, wordt u gevraagd te beslissen over deelname aan dit onderzoek. Indien u toestemming geeft, zullen wij u vragen deze op de bijbehorende toestemmingsverklaring schriftelijk te bevestigen. Door uw schriftelijke toestemming geeft u aan dat u

de informatie heeft begrepen en instemt met deelname van uw naaste aan het onderzoek. Het handtekeningenblad wordt door de onderzoeker bewaard. U krijgt een kopie of een tweede exemplaar van deze toestemmingsverklaring.

Dank voor uw tijd.

1. **Bijlagen bij deze informatie**

A. Contactgegevens

B. Informatie over de verzekering

C. Overzicht onderzoekshandelingen

D. Toestemmingsformulier

**Bijlage A: contactgegevens**

Als u nog vragen heeft over dit onderzoek, neemt u dan gerust contact op met een van de onderstaande onderzoeksmedewerkers:

**Coördinerend onderzoeker** Julia van der A, MSc

Heidelberglaan 100, 3584 CX Utrecht

j.vandera-2@umcutrecht.nl

Tel. (088) 756 75 23

**Onderzoeker UMC Utrecht:** Prof. dr. Arjen J.C. Slooter

Heidelberglaan 100, 3584 CX Utrecht

A.Slooter-3@umcutrecht.nl

**Onderzoeker Radboudumc:** Prof. dr. Indira Tendolkar

Reinier Postlaan 10, 6525 GC Nijmegen

Indira.Tendolkar@radboudumc.nl

**Onderzoeker HagaZiekenhuis:**  Dr. Thomas Ottens

Els Borst-Eilersplein 275, 2545 AA Den Haag

T.Ottens@hagaziekenhuis.nl

**Onafhankelijk arts:**  Dr. Maarten M.J. van Eijk, Intensivist-anesthesioloog

Afdeling Intensive Care UMC Utrecht

m.m.j.vaneijk-7@umcutrecht.nl

**Klachten:**

Als u klachten heeft kunt u dit melden aan de onderzoeker of aan uw behandelend arts. Mocht u ontevreden zijn over de gang van zaken bij het onderzoek en een klacht willen indienen dan kunt u contact opnemen met de klachtenbemiddelaars van het ziekenhuis. Heeft u vragen/klachten over de toepassing en naleving van de privacyregels, dan kunt u terecht bij de functionaris gegevensbescherming.

**UMC Utrecht**

De klachtenbemiddelaar is beschikbaar via tel. 088-755 62 08. Of digitaal via: <http://www.umcutrecht.nl/nl/Ziekenhuis/Ervaringen-van-patienten/Een-klacht-indienen>

**Contactgegevens Functionaris voor de Gegevensbescherming:**

post: UMC Utrecht t.a.v. Functionaris gegevensbescherming

Huispostnummer Fac. 10.12

Postbus 85500 3508GA Utrecht

e-mail: privacy@umcutrecht.nl

Voor meer informatie over uw rechten:

Raadpleeg de website van het UMC Utrecht voor meer informatie over uw rechten: <https://www.umcutrecht.nl/nl/Ziekenhuis/In-het-ziekenhuis/Regels-en-rechten/Rechten>

**HagaZiekenhuis**

De klachtenfunctionarissen zijn bereikbaar via e-mail of post.

E-mail: klachten.suggesties@hagaziekenhuis.nl

Post: HagaZiekenhuis Den Haag, t.a.v. klachtenfunctionaris

Antwoordnummer 1320

2504 VB Den Haag (een postzegel is niet nodig)

U kunt uw klacht ook indienen via een digitaal formulier. Ga hiervoor naar: <https://www.hagaziekenhuis.nl/over-hagaziekenhuis/ook-goed-om-te-weten/klachten-en-suggesties/klachtenformulier/>

**Contactgegevens** **Functionaris voor de Gegevensbescherming:**

E-mail: fg@hagaziekenhuis.nl

Telefoon: 070-210 0000 en vraag om doorverbonden te worden met de functionaris gegevensbescherming.

**Radboudumc**

De klachtenbemiddelaar is beschikbaar via: tel. (024) 361 31 91 of per post:

Radboudumc

348 Afdeling Klachtenbemiddeling

Antwoordnummer 540

6500 VC Nijmegen

U kunt uw klacht ook indienen via een digitaal formulier. Ga hiervoor naar: <https://www.radboudumc.nl/patientenzorg/uw-afspraak/meer-informatie/klachten/vier-stappen-om-een-klacht-in-te-dienen/klachtenbemiddelaar>

**Contactgegevens Functionaris voor de Gegevensbescherming**

Radboudumc

t.a.v. Functionaris voor Gegevensbescherming

Huispost 27

Postbus 9101

6500 HB NIJMEGEN

Website Privacy: https://www.radboudumc.nl/patientenzorg/rechten-en-plichten/privacy

E-mail: gegevensbescherming@radboudumc.nl

**Bijlage B: informatie over de verzekering**

Voor iedereen die meedoet aan dit onderzoek, heeft het UMC Utrecht een verzekering afgesloten. De verzekering dekt schade door deelname aan het onderzoek. Dit geldt voor schade tijdens het onderzoek of binnen vier jaar na het einde van deelname aan het onderzoek. Schade moet binnen die vier jaar aan de verzekeraar zijn gemeld.

De verzekering dekt niet alle schade. Onderaan deze tekst staat in het kort welke schade niet wordt gedekt. Deze bepalingen staan in het 'Besluit verplichte verzekering bij medisch-wetenschappelijk onderzoek met mensen 2015'. Dit besluit staat in de Wettenbank van de overheid (<https://wetten.overheid.nl>).

Bij schade kunt u direct contact leggen met de verzekeraar.

De verzekeraar van het onderzoek is:

Naam verzekeraar: QBE Europe SA/NV Nederland

Adres: Prins Bernhardplein 200, 1097 JB, Amsterdam

Telefoonnummer: +31 (0)20 899 2700

E-mail: info@nl.qbe.com

Polisnummer: 064457/01/2023/0000

De verzekering betaalt maximaal € 650.000 per persoon en € 5.000.000 voor het hele onderzoek € 7.500.000 per jaar voor alle onderzoeken van dezelfde opdrachtgever.

Let op: de verzekering dekt de volgende schade **niet**:

- Schade door een risico waarover we u informatie hebben gegeven in deze brief. Maar dit geldt niet als het risico groter bleek te zijn dan we van tevoren dachten. Of als het risico heel onwaarschijnlijk was.
- Schade aan de gezondheid van uw naaste die ook zou zijn ontstaan als u niet aan het onderzoek had meegedaan.
- Schade die ontstaat doordat uw naaste aanwijzingen of instructies niet of niet goed opvolgde.
- Schade aan de gezondheid van de kinderen of kleinkinderen van uw naaste.
- Schade door een behandelmethode die al bestaat. Of door onderzoek naar een behandelmethode die al bestaat.

**Bijlage C: Overzicht procedures DELTES-studie**

| **Procedures (tijd)** | **Baseline visite (V1)** | **Eerste behandeling (T1)** | **Vervolg behandeling (dag 2 tot dag 14^a^)** | **Eind van de behandeling (V2)** | **Telefonische visite (V3)** |
| --- | --- | --- | --- | --- | --- |
| Vragenlijsten delier (5-15 min) | X | X | X | X |  |
| tACS (30 min) |  | X | X |  |  |
| EEG (40 min) | X^b^ | X |  | X |  |
| Vragenlijsten ervaring behandeling (10 min) |  | X |  | X |  |
| Telefonische vragenlijst cognitie (15 min) |  |  |  |  | X |
| **Verwachte tijdsduur** | 15 of 55 min | 95 min | 45 min | 65 min | 15 min |

EEG = Electroencephalogram; tACS = behandeling met hersenstimulatie

^a^ = afhankelijk of uw naaste nog delirant is

^b^ = dit geldt alleen voor de patiënten die de gepersonaliseerde behandeling krijgen

**Bijlage D: Toestemmingsformulier vertegenwoordiger, DELTES-studie**

Horende bij: Een studie naar het effect van elektrische hersenstimulatie bij mensen met delirium (acute verwardheid), DELTES-studie

Naam proefpersoon: ……………………........................... Geb. datum: ………………………….

- Ik heb de informatiebrief voor de proefpersoon/vertegenwoordiger gelezen. Ook kon ik vragen stellen. Mijn vragen zijn goed genoeg beantwoord. Ik had genoeg tijd om te beslissen of ik wil dat deze persoon meedoet.
- Ik weet dat meedoen vrijwillig is. Ook weet ik dat ik op ieder moment kan beslissen dat deze persoon toch niet meedoet. Ik hoef dan niet te zeggen waarom ik dat wil.
- Ik geef de onderzoeker toestemming om de behandelend arts/specialist te laten weten dat deze persoon meedoet aan dit onderzoek.
- Ik geef de onderzoeker toestemming om de specialist van deze persoon informatie te geven over onverwachte uitkomsten van het onderzoek die van belang zijn voor de gezondheid van deze persoon.
- Ik geef de onderzoekers toestemming om de gegevens van deze persoon te verzamelen en te gebruiken. De onderzoekers doen dit om alleen de onderzoeksvraag in dit onderzoek te beantwoorden.
- Ik weet dat voor de controle van het onderzoek sommige mensen toegang tot alle gegevens van deze persoon kunnen krijgen. Die mensen staan in deze informatiebrief. Ik geef deze mensen toestemming om de gegevens van deze persoon in te zien voor deze controle.
- Ik geef **☐ wel / ☐ geen*** toestemming voor het doorsturen van gecodeerde gegevens van deze persoon in het kader van ander onderzoek naar landen buiten de EU. Ik weet dat buiten de EU mogelijk andere privacy regels gelden. Ik weet dat er voor het delen van de gegevens een gelijkwaardig beschermingsniveau zal worden afgesproken.
- Ik geef **☐ wel / ☐ geen*** toestemming om de gegevens van deze persoon te bewaren om die te gebruiken voor ander onderzoek, zoals in de informatiebrief staat.
- Ik geef **☐ wel / ☐ geen*** toestemming om deze persoon na dit onderzoek te vragen of hij/zij wil meedoen met een vervolgonderzoek
- Ik ga ermee akkoord dat deze persoon meedoet aan dit onderzoek.

Naam wettelijk vertegenwoordiger:……………………………

Relatie tot de proefpersoon: ……………………………………

Handtekening: ………………………………… Datum: _____ / _____ / _____

-------------------------------------------------------------------------------------------------------------------------------------

Ik verklaar dat ik de persoon/personen hierboven volledig heb geïnformeerd over het genoemde onderzoek.

Wordt er tijdens het onderzoek informatie bekend die de toestemming van de vertegenwoordiger kan beïnvloeden? Dan laat ik dit op tijd aan deze persoon weten.

Naam onderzoeker (of diens vertegenwoordiger):………………………....................................

Handtekening: ………………………………… Datum: _____ / _____ / _____

--------------------------------------------------------------------------------------------------------------------------------------

* Aankruisen wat van toepassing is.

*De vertegenwoordiger krijgt een volledige informatiebrief mee, samen met een getekende versie van het toestemmingsformulier.*
