# Supplementary material for: DELirium treatment with Transcranial Electrical Stimulation (DELTES): study protocol for a multicentre, randomised, double-blind, sham-controlled trial
Source: BMJ Open. 2024 Nov 2;14(11):e092165. doi: 10.1136/bmjopen-2024-092165 (PMC11535714; doi:10.1136/bmjopen-2024-092165)
Supplement: Supplementary file 3 [file bmjopen-2024-092165.reviewer_comments.pdf]

PEER REVIEW HISTORY

BMJ Open publishes all reviews undertaken for accepted manuscripts. Reviewers are asked to complete a checklist review form and are provided with free text boxes to elaborate on their assessment. These free text comments are reproduced below.

ARTICLE DETAILS

Title (Provisional)

DELirium treatment with Transcranial Electrical Stimulation (DELTES): study protocol for a multicentre, randomised, double-blind, sham-controlled trial

Authors

van der A, Julia; Lodema, Yorben; Ottens, Thomas H.; Schutter, D.J.L.G.; Emmelot-Vonk, Marielle H.; de Haan, Willem; van Dellen, Edwin; Tendolkar, Indira; Slooter, Arjen J.C

VERSION 1 - REVIEW

Reviewer 1

Name Lei, Chong

Affiliation Fourth Military Medical University, Department of Anesthesiology and Periioprative Medicine, Xijing Hospital

Date 26-Aug-2024

COI None

The authors designed a randomized, 2 phases, double-blinded study to investigated if the tACS is effective in treating delirium.

1.It was a two-phase study, in the pilot study the feasibility of tACS will be tested, and the data of the pilot study will be used in the final analysis, which made this seemed to be a seamless study. However, how the result of the pilot study will be used to inform the main study did not described thorough. In addition, a new arm of personalized tACS was introduced in the main study with a new sham procedure of the personalized tACS. These arises the concern that why the safety and feasibility of this personalized tACS not need to validated in the pilot study. If different strategies are aimed to investigated in one trial, how about to design a platform trial.

2.The hypothesis of this study was not clearly declaimed in the protocol. If both of the personalized and standardize tACS are effective, them why bother to use the personalized strategy. What is the hypothesis regarding the personalized strategy. In addition, different shams are set up and then merge to a sham group. Why design like this? If the sham procedure are different, then it is better not to merge these two groups.

3.The target population of interests was patients with diagnosed delirium for at least 2 days with underlying causes adequately treated. It is hard to confirm if the underlying causes were adequately treated. Too much restriction will jeopardize the generalizablity of the study.

4.Both surgical and non-surgical population will be enrolled. The mechanism and underlying causes are different, which introduces heterogeneous. In addition, postoperative delirium is considered as a self-limiting disease, only small proportion of them will have symptoms persisted for a long time. Then, use of tACS, a relative invasive treatment may not be ethical.

5.The primary endpoints was the change of delta wave. However, it is just a surrogate endpoint, I suggest the clinical relevant endpoints, such as delirium resolution, duration, or severity. What is more, as reported in the Br J Anaesth. 2019;122(1):60-68, "The likelihood of delirium , severity of delirium , attentional level, and level of consciousness were all signifificantly, but weakly, correlated with both the relative delta power 1-4 Hz and the relative power from 1 to 6 Hz." To my opinion, delta power was not even a good surrogate endpoint.

6.There is no any evidence indicated the 0.15 effect size for sample size calculation is reasonable.

**Reviewer**	**2**
**Name**	**Leroy, Sophie**
**Affiliation**	**Charite - Universitatsmedizin Berlin**
**Date**	**05-Sep-2024**
**COI**	**No competing interests to declare**

The results of this trial will represent a significant milestone in the field and could profoundly impact the care and management of patients with delirium. Especially when all pharmaceutical and non-pharmaceutical intervention do not show any effect, innovative therapeutical concepts are lacking. Overall it is a well written protocol paper. The trial is highly ambitious given the in comparison relatively small sample size in a very heterogenous population, which poses a risk of not achieving statistically significant results. However, these concerns do not preclude the publication of the protocol paper in any way. Here are a few comments for the authors:

1. Frequency of Visits and Delirium Assessments: It seems surprising that the study protocol plans for only once-daily visits and delirium assessments, particularly in the pilot phase and especially since some of the authors have co-authored a consensus stating that delirium should be monitored at least once per shift. Given that this is the first trial evaluating the effects of tACS on patients with delirium, fluctuations in arousal or attention levels due to

tACS could be a conceivable effect. To ensure adequate monitoring of safety, once-daily assessments may be insufficient.

2. Questionnaire for Adverse Events and Treatment Experience: Interpreting a questionnaire to assess subjective treatment experiences in patients with an acute disorder of consciousness and attention seems challenging.

3. Sample Size Estimation: Regarding the sample size calculation, it appears that a Bonferroni correction for multiple testing may not have been applied. Based on the parameters provided, G*Power suggests a required sample size of 63 patients per group for $p < 0.025$, and 53 patients for $p < 0.05$. Please confirm whether this correction has been applied or revise the estimation accordingly.

4. Attrition Considerations: The statement "As the primary outcome assessment occurs right after the first treatment, no attrition is expected" is surprising. Even during the first treatment, dropouts due to withdrawal of consent, failure to re-consent, or adverse events should be anticipated. The authors note that patients with delirium could be too agitated to undergo EEG recordings, and early termination of the first stimulation session could also result in missing primary outcome data.

5. Heterogeneous Study Population: The study includes a highly heterogeneous population with different delirium etiologies (e.g., medical vs. POD, ICU vs. non-ICU, cardiac vs. non-cardiac surgery, patients with and without concomitant neuropsychiatric diseases, patients with and without concomitant centrally-acting medication…). This diversity could impact the study results and should be considered in the analysis and interpretation of data.

6. Study Design Concerns: While personalized tACS is a promising approach in tES research, it may be premature to combine a feasibility trial, a pilot study, and an individualized treatment approach in a single study. It is unclear how these steps can be effectively integrated into a single trial with a cohort of max. 53 very heterogeneous patients, accounting for potential dropouts. It seems like this project was part of some kind of grant proposal where the authors are affiliated. If I understand it correctly this is the reason for this specific study design.

7. Alternatives: It seems a bit hasty to publish a protocol for the main phase of the trial before the modelling of the tACS effect on a delirious network has been performed. Consider mentioning an alternative approach for the personalized tACS, in case no conclusive results arise from modelling.

8. tACS Artifacts in ECG Monitoring: tACS artifacts may appear in ECG monitoring, particularly in the ICU patients. It would be beneficial to mention a strategy to avoid unblinding due to these artifacts.

9. Primary Endpoint Selection: While I understand the choice of an EEG parameter as the primary outcome for this trial, I was surprised that the authors did not choose a coherence measure. By selecting "relative delta power" as the primary outcome, the study's results

may be difficult to interpret in terms of clinical relevance. Changes in relative delta power could result from either oscillatory entrainment (synchronization of brain wave activity) or a rebound phenomenon (a temporary effect that does not necessarily translate into lasting clinical improvements).

VERSION 1 - AUTHOR RESPONSE

For a point-by-point response, please see the attached rebuttal letter.

Reviewer #1
Prof. Chong Lei, Fourth Military Medical University
Comments to the Author:
The authors designed a randomized, 2 phases, double-blinded study to investigated if the tACS is effective in treating delirium.

1.It was a two-phase study, in the pilot study the feasibility of tACS will be tested, and the data of the pilot study will be used in the final analysis, which made this seemed to be a seamless study. However, how the result of the pilot study will be used to inform the main study did not described thorough. In addition, a new arm of personalized tACS was introduced in the main study with a new sham procedure of the personalized tACS. These arises the concern that why the safety and feasibility of this personalized tACS not need to validated in the pilot study. If different strategies are aimed to investigated in one trial, how about to design a platform trial.

Author's response:
Thank you for your insightful comments. We appreciate the opportunity to clarify our study design and rationale. Our two-phase approach aims to streamline the research process while ensuring the feasibility of tACS in our target population. The pilot study primarily tests feasibility and allows us to refine study procedures, but we do not anticipate major alterations to the study design after this phase. Regarding the personalised tACS arm, its introduction in the main study is based on the assumption that personalisation would optimise therapeutic outcomes rather than introduce new risks. The personalisation affects stimulation parameters within pre-established safe ranges, and the output of the personalised model falls well within the safety parameters for tACS stimulation. This means that any treatment based on the computational model's input is safe, and the model itself cannot influence treatment safety.

While we acknowledge that validating personalised tACS in the pilot phase could provide additional insights, we believe our current approach balances scientific rigor with practical considerations such as time constraints.

Regarding the suggestion of a platform trial, we appreciate the recommendation. However, our current design aligns well with our specific research questions and available resources. Our primary aim is to compare both standardised and personalised tACS with sham stimulation on relative delta power, which can be effectively accomplished with our proposed design. We will certainly consider a platform trial design for future, larger-scale studies investigating multiple tACS strategies.

2.The hypothesis of this study was not clearly declaimed in the protocol. If both of the personalized and standardize tACS are effective, them why bother to use the personalized strategy. What is the hypothesis regarding the personalized strategy. In addition, different shams are set up and then merge to a sham group. Why design like this? If the sham procedure are different, then it is better not to merge these two groups.

Author's response: We agree with the reviewer that the hypothesis regarding personalised and standardised tACS is not clearly stated in the manuscript. Our hypothesis is that the personalised treatment strategy is superior compared to standardised tACS in reducing relative delta power. However, as there is no data available on tACS in delirious patients and its effect on EEG, we currently lack data to substantiate this claim and calculate the effect size accordingly. Therefore, we calculated the sample size based on the assumption that both treatment arms have equal effectiveness. We have added a sentence to further clarify our hypothesis:

Methods and analysis (p. 8): "<u>We hypothesise that personalised tACS may be superior to standardised tACS in reducing relative delta power. However, the lack of data to support this claim necessitates assuming equal effectiveness for both arms in the sample size calculation."</u>

Regarding the design involving two sham groups, we require distinct sham conditions for both the personalised tACS and the sham personalised tACS to ensure that participants remain unaware of their group allocation (active personalised tACS or sham personalised tACS). Although the two sham procedures differ minimally, we are confident that merging them into a single sham group is appropriate due to their similarities. This consolidation allows us to reduce the overall number of participants needed, thereby enhancing the feasibility of the study while minimising the burden on patient recruitment.

3.The target population of interests was patients with diagnosed delirium for at least 2 days with underlying causes adequately treated. It is hard to confirm if the underlying causes were adequately treated. Too much restriction will jeopardize the generalisability of the study.

Author's response: We appreciate the reviewer's concern about balancing thorough assessment of underlying causes with maintaining generalizability. We agree that confirming adequate treatment of all underlying causes is challenging. Our intention with this inclusion criteria is to ensure patients receive appropriate basic standard of care for identifiable underlying conditions before enrolling in the study, without overly restricting our sample.

4. Both surgical and non-surgical population will be enrolled. The mechanism and underlying causes are different, which introduces heterogeneous. In addition, postoperative delirium is considered as a self-limiting disease, only small proportion of them will have symptoms persisted for a long time. Then, use of tACS, a relative invasive treatment may not be ethical.

Author's response:
We appreciate the reviewer's thoughtful comments regarding the heterogeneity of our study population and the ethical considerations of using tACS for postoperative delirium. Regarding population heterogeneity, recent work by our group (Lodema et al., 2024) found that EEG variables could not distinguish between postoperative delirium and non-surgical delirium. This suggests that the underlying neurophysiological changes in delirium are similar across different aetiologies. In addition, numerous factors that increase the risk of postoperative delirium, are risk factors for non-postoperative delirium as well, examples are advanced age, opioid use and infectious diseases.

We respectfully disagree with the characterisation of postoperative delirium as uniformly self-limiting. While some cases may resolve quickly, numerous studies indicate that prolonged (postoperative) delirium is associated with significant adverse outcomes (Aung Thein et al., 2020; Bellelli et al., 2014; Kirfel et al., 2022). Additionally, our extensive combined clinical experience is that, especially after complex surgery such as cardiothoracic surgery, delirium may persist for days to

weeks in a significant portion of patients (140/1599) (Kooken et al., 2021). Given the potentially severe consequences, and considering that we only include patients who have been delirious for at least two days, we believe that exploring tACS as a treatment option is ethically justified. Furthermore, tACS is generally well-tolerated with few, mild side effects and is considered a non-invasive treatment. Most reported symptoms show no difference between active and sham stimulation, and any side effects are typically mild and short-lived. We believe that the potential benefits of finding an effective treatment for prolonged delirium outweigh the minimal risks associated with tACS, especially given the significant long-term impact on cognition and quality of life associated with persistent delirium.

5.The primary endpoints was the change of delta wave. However, it is just a surrogate endpoint, I suggest the clinical relevant endpoints, such as delirium resolution, duration, or severity. What is more, as reported in the Br J Anaesth. 2019;122(1):60-68, “The likelihood of delirium , severity of delirium , attentional level, and level of consciousness were all significantly, but weakly, correlated with both the relative delta power 1-4 Hz and the relative power from 1 to 6 Hz.” To my opinion, delta power was not even a good surrogate endpoint.

Author’s response: As this is the very first study investigating tACS in delirium, our primary interest is to determine whether tACS can induce neurophysiological changes indicative of a less delirious EEG state. Relative delta power has been consistently shown to be elevated in delirium across various populations (Boord et al., 2021) and has been demonstrated to effectively classify patients as delirious (Numan et al., 2019), making it a reliable marker of delirium-associated EEG changes. We recognize the importance of clinically relevant endpoints, as highlighted by the reviewer. To address this, we have included several clinical variables as secondary outcomes, including delirium resolution, duration, and severity. The clinical significance of changes in relative delta power will be evaluated in conjunction with these secondary outcomes, providing a comprehensive assessment of the intervention's impact.

6.There is no any evidence indicated the 0.15 effect size for sample size calculation is reasonable.

Author’s response: Our sample size calculation is based on a previous study that obtained data in both delirious and non-delirious patients. Patients with delirium showed a median relative delta power of 0.59 (interquartile range (IQR) 0.47-0.71), while those without delirium had a median of 0.20 (IQR 0.17-0.26), resulting in a raw mean difference of 0.39. As we expect our study to have more heterogeneity than this sample, a conservative but realistic estimate would be a decrease of 0.15 in relative delta EEG power post-stimulation compared to pre-stimulation measurements. We agree with the reviewer that this is not further supported by scientific evidence, however, as this is the first study to investigate the effect of tACS on relative delta power in delirious patients, such data does not (yet) exist.

Reviewer #2

Dr. Sophie Leroy, Charite - Universitatsmedizin Berlin
Comments to the Author:
The results of this trial will represent a significant milestone in the field and could profoundly impact the care and management of patients with delirium. Especially when all pharmaceutical and non-pharmaceutical intervention do not show any effect, innovative therapeutical concepts are lacking. Overall it is a well written protocol paper. The trial is highly ambitious given the in comparison relatively small sample size in a very heterogenous population, which poses a risk of not achieving statistically significant results. However, these concerns do not preclude the

publication of the protocol paper in any way. Here are a few comments for the authors:

Author's response: We thank the reviewer for the positive and constructive feedback on our manuscript. Below a point-by-point response is presented:

1. Frequency of Visits and Delirium Assessments: It seems surprising that the study protocol plans for only once-daily visits and delirium assessments, particularly in the pilot phase and especially since some of the authors have co-authored a consensus stating that delirium should be monitored at least once per shift. Given that this is the first trial evaluating the effects of tACS on patients with delirium, fluctuations in arousal or attention levels due to tACS could be a conceivable effect. To ensure adequate monitoring of safety, once-daily assessments may be insufficient.

Author's response: We appreciate the reviewer's important observation regarding the frequency of visits and delirium assessments in our study protocol. The decision to conduct once-daily delirium assessments was made to minimize patient burden and consider staffing limitations. To ensure adequate monitoring of safety, we screen the electronic patient record and consult with the treating physician or nurse about any health changes, such as increases in antipsychotic medication, since the previous tACS session. Any event potentially related to the study procedures will be classified as an AE. This was not yet described in the manuscript. To address this, we have added the following sentence:

Methods and analysis (p. 18): "On each treatment day, the study team will screen the electronic patient record and consult with the treating physician or nurse about any health changes since the previous tACS session. Any event potentially related to the study procedures will be classified as an AE."

We acknowledge the potential for fluctuations in delirium symptoms, particularly in the context of tACS treatment. To address this concern, we incorporate information from nursing staff when scoring the Intensive Care Delirium Screening Checklist (ICDSC), especially with regard to the "symptom fluctuation" question. Additionally, to account for fluctuations in delirium, we consider a delirious episode to have resolved only after obtaining two consecutive negative delirium assessments, rather than one. This was not yet described in the manuscript, and to address this we have added the following sentence to the manuscript:

Methods and analysis (p.7): "To account for fluctuations in delirium symptoms, resolution of delirium is defined as two consecutive negative delirium assessments."

2. Questionnaire for Adverse Events and Treatment Experience: Interpreting a questionnaire to assess subjective treatment experiences in patients with an acute disorder of consciousness and attention seems challenging.

Author's response: We acknowledge that this presents a significant challenge and we recognize that many patients may not be able to provide answers. However, as tACS has not previously been applied to delirious patients, the perspectives of patients who can answer offers important information. We have designed our feasibility questions to be posed to nursing staff and family members present during the initial treatment session. This strategy aims to capture relevant feedback while the patient may be unable to engage meaningfully. Furthermore, we re-administer the remaining subjective questionnaires at the close-out visit (V2) when the patient is no longer in a state of delirium, allowing for more accurate and insightful responses.

3. Sample Size Estimation: Regarding the sample size calculation, it appears that a Bonferroni correction for multiple testing may not have been applied. Based on the parameters provided, G*Power suggests a required sample size of 63 patients per group for p < 0.025, and 53 patients for p < 0.05. Please confirm whether this correction has been applied or revise the estimation accordingly.

Author's response: We appreciate the reviewer's careful attention to our sample size calculation and the point raised about multiple testing correction. The reviewer is correct in noting that we did not apply a Bonferroni correction in our initial sample size estimation. This was not stated correctly in our manuscript. As this is the first study of its kind, we opted for an exploratory analysis approach and therefore to use a less stringent significance level to avoid missing potentially important effects that could be further investigated in future confirmatory studies. We have edited the manuscript accordingly on the following pages:

Methods and analysis (p. 9): "adjusted for multiple testing using Bonferroni correction" has been removed.
Methods and analysis (p.20): "to correct for type I erros since there are two intervention groups (standardised and personalised tACS)" has been removed.

The following sentence has been added:
Methods and analysis (p.20): "To retain sensitivity to detect potential effects in this novel area of research, no adjustment for multiple comparisons will be made."

4. Attrition Considerations: The statement "As the primary outcome assessment occurs right after the first treatment, no attrition is expected" is surprising. Even during the first treatment, dropouts due to withdrawal of consent, failure to re-consent, or adverse events should be anticipated. The authors note that patients with delirium could be too agitated to undergo EEG recordings, and early termination of the first stimulation session could also result in missing primary outcome data.

Author's response: The reviewer has raised a valid point about the potential for dropouts. For our primary outcome, we will employ a per-protocol analysis, which stipulates that participants must have undergone at least the first tACS session including EEG directly before and after to be included in the analysis. Importantly, participants will not be excluded for missing tACS sessions after the initial treatment. To maintain sufficient statistical power, patients who did not complete the initial tACS session with EEG recordings will be replaced as well as patients who withdrawal consent. We recognize that this approach was not adequately conveyed in the original manuscript. To enhance clarity, we have revised the relevant sections of the manuscript to emphasise this.

Methods and analysis (p. 9): "As the primary outcome assessment occurs right after the first treatment, no attrition is expected." has been removed.

The following sentences/parts has been added:

Methods and analysis (p. 9): "Patients who do not complete the initial tACS session with EEG recordings will be replaced, as well as patients who withdraw consent.

Methods and analysis (p. 17). "For the analysis of the primary study parameter, a per-protocol analysis will be used. The sole criterion for inclusion in the analysis is that a participant has completed the initial tACS session and EEG recordings.

5. Heterogeneous Study Population: The study includes a highly heterogeneous population with different delirium etiologies (e.g., medical vs. POD, ICU vs. non-ICU, cardiac vs. non-cardiac surgery, patients with and without concomitant neuropsychiatric diseases, patients with and without concomitant centrally-acting medication…). This diversity could impact the study results and should be considered in the analysis and interpretation of data.

Author's response: We thank the reviewer for this observation regarding the heterogeneity of our study population. We acknowledge that this diversity can indeed present challenges and potentially impact our study results. However, we believe this heterogeneous sample closely reflects the diverse patient groups typically encountered in clinical practice, thereby enhancing the external validity and generalizability of our findings.

Regarding the primary outcome, recent work published by our group (Lodema et al., 2024) found that EEG variables could not distinguish between postoperative delirium and other types of delirium (metabolic, infectious) in a sample of 129 patients experiencing delirium. This suggests that the underlying neurophysiological changes in delirium may be consistent across different aetiologies.

Nevertheless, we agree with the reviewer that the diversity should be carefully considered in the analysis and interpretation of our data. To address this, we have expanded our analytical approach:

Methods and analysis (p.18): "Subgroup analysis will be conducted by including additional fixed factors to the mixed models, such as delirium aetiology, sex and age."

6. Study Design Concerns: While personalized tACS is a promising approach in tES research, it may be premature to combine a feasibility trial, a pilot study, and an individualized treatment approach in a single study. It is unclear how these steps can be effectively integrated into a single trial with a cohort of max. 53 very heterogeneous patients, accounting for potential dropouts. It seems like this project was part of some kind of grant proposal where the authors are affiliated. If I understand it correctly this is the reason for this specific study design.

Author's response: We appreciate the reviewer's thoughtful insights regarding the study design. We acknowledge the complexity of integrating a feasibility trial, pilot study, and individualised treatment approach within a single study. However, participants that are part of the pilot and feasibility phase will be combined in the main study phase. Therefore, this design allows us to efficiently assess applicability of (personalised) tACS in delirious patients. We believe the issues raised concerning the heterogeneous nature of the study and potential drop-out of participants were already answered under comment 4 and 5, respectively.

7. Alternatives: It seems a bit hasty to publish a protocol for the main phase of the trial before the modelling of the tACS effect on a delirious network has been performed. Consider mentioning an alternative approach for the personalized tACS, in case no conclusive results arise from modelling.

Author's response: The reviewer has pointed out an important question relating to our study design of the personalised treatment arm. In line with the suggestion by the reviewer, we have added a more thorough explanation of the possible directions we are exploring to fit our model to individual EEG characteristics. These range from straightforward and less sophisticated (individual peak

frequency fit) to more complex (disease model fit on multiple dimensions), ensuring a feasible outcome to apply in the trial. We have added the following sentences to address this point:

Methods and analysis (p. 14): In this phase, several strategies will be considered: a disease model tailored at multiple dimensions to the individual neurophysiology (de Haan et al., 2017), a model tailored to the individual peak frequency (Fresnoza et al., 2018), or spatial modelling of individual brain activity. The results of this development process will be published in a separate paper describing the details of this approach and the most effective strategy will be utilised in the second phase of the trial.

8. tACS Artifacts in ECG Monitoring: tACS artifacts may appear in ECG monitoring, particularly in the ICU patients. It would be beneficial to mention a strategy to avoid unblinding due to these artifacts.

Author's response: We thank the reviewer for addressing this. We are aware of the possibility of tACS artifacts affecting ECG monitoring, especially in ICU settings. We therefore mask the ECG monitor before starting the stimulation, but the reviewer is correct that this was not mentioned in the manuscript. We have added this to the manuscript as follows:

Methods and analysis (p.11): "To ensure blinding during the intervention, the monitor displaying the raw ECG traces will be covered with cardboard paper before the start of the procedure for patients on continuous ECG monitoring."

9. Primary Endpoint Selection: While I understand the choice of an EEG parameter as the primary outcome for this trial, I was surprised that the authors did not choose a coherence measure. By selecting "relative delta power" as the primary outcome, the study's results may be difficult to interpret in terms of clinical relevance. Changes in relative delta power could result from either oscillatory entrainment (synchronization of brain wave activity) or a rebound phenomenon (a temporary effect that does not necessarily translate into lasting clinical improvements).

Author's response:
We agree with the reviewer that coherence measures may offer valuable insights. However, given that the effects of tACS on EEG parameters in patients with delirium are not yet understood, we believe that it is essential to utilize a parameter that has consistently demonstrated alteration during episodes of delirium. We understand the concern about interpreting clinical relevance, but we believe this applies equally to coherence measures. The relationship between EEG changes and clinical improvement remains unclear for both measures. The clinical significance of changes in relative delta power will be evaluated alongside our secondary outcomes, including delirium duration and length of hospital stay, which will provide a comprehensive assessment of the intervention's impact. Furthermore, we will analyse other qEEG characteristics (including coherence measures) as secondary outcomes, as stated in the methods and materials.

References

Aung Thein, M. Z., Pereira, J. V., Nitchingham, A., & Caplan, G. A. (2020). A call to action for delirium research: Meta-analysis and regression of delirium associated mortality. *BMC Geriatrics*, *20*(1). https://doi.org/10.1186/s12877-020-01723-4

Bellelli, G., Mazzola, P., Morandi, A., Bruni, A., Carnevali, L., Corsi, M., Zatti, G., Zambon, A., Corrao, G., Olofsson, B., Gustafson, Y., & Annoni, G. (2014). Duration of postoperative delirium is an

independent predictor of 6-month mortality in older adults after hip fracture. *Journal of the American Geriatrics Society*, *62*(7), 1335–1340. https://doi.org/10.1111/jgs.12885
Boord, M. S., Moezzi, B., Davis, D., Ross, T. J., Coussens, S., Psaltis, P. J., Bourke, A., & Keage, H. A. D. (2021). Investigating how electroencephalogram measures associate with delirium: A systematic review. In *Clinical Neurophysiology* (Vol. 132, Issue 1, pp. 246–257). Elsevier Ireland Ltd. https://doi.org/10.1016/j.clinph.2020.09.009
de Haan, W., van Straaten, E. C. W., Gouw, A. A., & Stam, C. J. (2017). Altering neuronal excitability to preserve network connectivity in a computational model of Alzheimer's disease. *PLoS Computational Biology*, *13*(9). https://doi.org/10.1371/journal.pcbi.1005707
Fresnoza, S., Christova, M., Feil, T., Gallasch, E., Körner, C., Zimmer, U., & Ischebeck, A. (2018). The effects of transcranial alternating current stimulation (tACS) at individual alpha peak frequency (iAPF) on motor cortex excitability in young and elderly adults. *Experimental Brain Research*, *236*(10), 2573–2588. https://doi.org/10.1007/s00221-018-5314-3
Kirfel, A., Guttenthaler, V., Mayr, A., Coburn, M., Menzenbach, J., & Wittmann, M. (2022). Postoperative delirium is an independent factor influencing the length of stay of elderly patients in the intensive care unit and in hospital. *Journal of Anesthesia*, *36*(3), 341–348. https://doi.org/10.1007/s00540-022-03049-4
Kooken, R. W. J., van den Berg, M., Slooter, A. J. C., Pop-Purceleanu, M., & van den Boogaard, M. (2021). Factors associated with a persistent delirium in the intensive care unit: A retrospective cohort study. *Journal of Critical Care*, *66*, 132–137. https://doi.org/10.1016/j.jcrc.2021.09.001
Lodema, D. Y., Ditzel, F. L., Hut, S. C. A., van Dellen, E., Otte, W. M., & Slooter, A. J. C. (2024). Single-channel qEEG characteristics distinguish delirium from no delirium, but not postoperative from non-postoperative delirium. *Clinical Neurophysiology*, *161*, 93–100. https://doi.org/10.1016/j.clinph.2024.01.009
Numan, T., van den Boogaard, M., Kamper, A. M., Rood, P. J. T., Peelen, L. M., Slooter, A. J. C., Abawi, M., van den Boogaard, M., Claassen, J. AHR., Coesmans, M., Dautzenberg, P., Dhondt, T. ADF., Diraoui, S. B., Eikelenboom, P., Emmelot-Vonk, M. H., Faaij, R. A., van Gool, W. A., Groot, E. R., Hagestein-de Bruijn, C., … van der Zwaag, S. (2019). Delirium detection using relative delta power based on 1-minute single-channel EEG: a multicentre study. *British Journal of Anaesthesia*, *122*(1), 60–68. https://doi.org/10.1016/j.bja.2018.08.021

VERSION 2 - REVIEW

Reviewer 1

Name Lei, Chong

Affiliation Fourth Military Medical University, Department of Anesthesiology and Perioprative Medicine, Xijing Hospital

Date 14-Oct-2024

COI

I currently have no further issues, as the revised manuscript has addressed my previous concerns.

**Reviewer**	**2**
**Name**	**Leroy, Sophie**
**Affiliation**	**Charite - Universitatsmedizin Berlin**
**Date**	**14-Oct-2024**
**COI**	

The authors have provided satisfactory responses to all the comments, in my opinion. I wish them success in completing the trial!
